# Host-Pathogen Interactions of *Mycoplasma mycoides* in Caprine and Bovine Precision-Cut Lung Slices (PCLS) Models

**DOI:** 10.3390/pathogens8020082

**Published:** 2019-06-20

**Authors:** Yenehiwot Berhanu Weldearegay, Sandy Müller, Jana Hänske, Anja Schulze, Aline Kostka, Nancy Rüger, Marion Hewicker-Trautwein, Ralph Brehm, Peter Valentin-Weigand, Robert Kammerer, Joerg Jores, Jochen Meens

**Affiliations:** 1 Institute for Microbiology, Department of Infectious Diseases, University of Veterinary Medicine Hannover, 30173 Hannover, Germany; Sandy.Mueller@tiho-hannover.de (S.M.); Anja.Schulze@tiho-hannover.de (A.S.); aline.kostka@tiho-hannover.de (A.K.); nancy.rueger@tiho-hannover.de (N.R.); Peter.Valentin@tiho-hannover.de (P.V.-W.); Jochen.Meens@tiho-hannover.de (J.M.); 2 Institute of Immunology, Friedrich-Loeffler-Institute, Federal Research Institute for Animal Health, 17493 Greifswald-Insel Riems, Germany; Jana.Haenske@lua.sms.sachsen.de (J.H.); Robert.Kammerer@fli.de (R.K.); 3 Institute for Pathology, University of Veterinary Medicine Hannover, 30559 Hannover, Germany; Marion.Hewicker-Trautwein@tiho-hannover.de; 4 Institute of Anatomy, University of Veterinary Medicine Hannover, 30173 Hannover, Germany; Ralph.Brehm@tiho-hannover.de; 5 Institute of Veterinary Bacteriology, University of Bern, CH-3001 Bern, Switzerland; joerg.jores@vetsuisse.unibe.ch; 6 International Livestock Research Institute, PO Box 30709, 00100 Nairobi, Kenya

**Keywords:** *Mycoplasma mycoides*, CBPP, MAKePS syndrome, PCLS, tropism, immunofluorescence, 3R

## Abstract

Respiratory infections caused by mycoplasma species in ruminants lead to considerable economic losses. Two important ruminant pathogens are *Mycoplasma mycoides* subsp. *Mycoides* (*Mmm*), the aetiological agent of contagious bovine pleuropneumonia and *Mycoplasma mycoides* subsp. *capri* (*Mmc*), which causes pneumonia, mastitis, arthritis, keratitis, and septicemia in goats. We established precision cut lung slices (PCLS) infection model for *Mmm* and *Mmc* to study host-pathogen interactions. We monitored infection over time using immunohistological analysis and electron microscopy. Moreover, infection burden was monitored by plating and quantitative real-time PCR. Results were compared with lungs from experimentally infected goats and cattle. Lungs from healthy goats and cattle were also included as controls. PCLS remained viable for up to two weeks. Both subspecies adhered to ciliated cells. However, the titer of *Mmm* in caprine PCLS decreased over time, indicating species specificity of *Mmm*. *Mmc* showed higher tropism to sub-bronchiolar tissue in caprine PCLS, which increased in a time-dependent manner. Moreover, *Mmc* was abundantly observed on pulmonary endothelial cells, indicating partially, how it causes systemic disease. Tissue destruction upon prolonged infection of slices was comparable to the in vivo samples. Therefore, PCLS represents a novel ex vivo model to study host-pathogen interaction in livestock mycoplasma.

## 1. Introduction

Mycoplasmas are small cell wall-less bacteria, belonging to the class of Mollicutes. Ruminant mycoplasma causes huge impacts on animal welfare and food production. The control of many ruminant mycoplasma depends on vaccines that are suboptimal. Five phylogenetically related mycoplasma species causing disease in ruminants are grouped as *Mycoplasma mycoides* (*M. mycoides*) cluster. The *M. mycoides* cluster comprises: *Mycoplasma mycoides* subspecies *mycoides* (*Mmm*), *M. mycoides* subsp. *capri* (*Mmc*), *M. capricolum* subsp. *capripneumoniae (Mccp)*, *M. capricolum* subsp. *capricolum* (*Mcc*), and *M. leachii* [1,2,3,4]. In this study, we focused on two members of *M. mycoides cluster* affecting cattle and goats, namely, *Mmm* and *Mmc,* respectively. 

*Mycoplasma mycoides* subsp. *mycoides* is the causative agent of contagious bovine pleuropneumonia (CBPP), a severe transboundary disease, which is notifiable to the World organization for animal health (OIE). It causes pneumonia associated with mortality, production losses, and trade restriction [5,6]. Clinical signs of the disease are attributable to lesions that develop in the thorax, where large areas of the lungs may be affected. In adult animals, the disease is characterized by fibrinous pneumonia and pleurisy, gray and red hepatization (marbling appearance) of the lung, sequestra formation, high accumulation of pleural effusion, fever, dyspnea, and loss of body condition [7]. Arthritis rather than pulmonary symptoms are characteristic features of the disease in affected calves [5,8]. In different parts of Africa, varying prevalence of CBPP was reported ranging from 4 to 63%, affecting the economies of these countries [9,10,11,12].

*Mmc* is the causative agent of mastitis, arthritis, keratitis, pneumonia, and septicemia (MAKePS) syndrome. The MAKePS syndrome occurs in goats and is caused by one species or a combination of *Mmc, M. capricolum* subsp. *capricolum, M. putrefaciens*, *and M. agalactiae* [13]. The prominent clinical sign in MAKePS syndrome is mastitis in lactating does, arthritis, and keratitis in adults; arthritis, pleuropneumonia, and septicemia in children. However, pulmonary tropism is mainly seen in disease associated with *Mmc* [13]. *Mmc* can also result in outbreaks with high mortality [14]. A prevalence of 24% was reported in Pakistan [15]. A study conducted in an artificial insemination center in Spain revealed an increase in the incidence of *Mmc* latent infection in goats [16]. Recently, an outbreak of *Mmc* was reported in the United States of America [17] indicating the importance of this pathogen and the threats posed regardless of the available control methods.

The control of CBPP and MAKePS syndrome mainly relies on vaccination of animals. Currently, the live vaccine strain T1/44 is used to control CBPP, however, it does not confer long-term immunity and occasionally causes adverse effects at the site of inoculation. In addition, inoculation of strain T1/44 via the endotracheal route still leads to CBPP, indicating that this vaccine strain is not efficiently attenuated [18,19]. Subunit vaccines have been developed for CBPP and show promising protection [20]. The disease spreads to new areas in Africa due to several factors such as inadequate funding of annual vaccination, shortage of vaccine [21], absence of cheap on farm screening test [22] and refusal of cattle owners to present their animals for vaccination due to post-vaccination adverse effects and other unknown reasons [23,24,25]. Candidate vaccine formulations against MAKePS have been tested [26] but such vaccines have not been tested in the field yet. In India, they tested different types of lyophilized saponified vaccines of *Mmc* and observed a protection up to 67% [27]. Better knowledge of host-pathogen interactions and pathogenesis will aid the development of novel rationale vaccines [19]. In the absence of a small animal model for most ruminant *Mycoplasma* species, there is a need to find suitable ex vivo or in vitro models, which are in line with 3R guidelines (replacement, reduction, and refinement) [28,29] to study host-pathogen interactions. In biomedical sciences, the use of three-dimensional (3D) organ models including organoids is becoming an intensive area of research due to the close similarity to host species [29]. One of the functional 3D organ models are precision cut lung slices (PCLS). As reported by Henjakovic and colleagues [30], PCLS has an advantage over other models, including isolated tracheal or bronchial rings, where the contraction is either isometric or isotonic. However, in PCLS, there is a parenchymal tethering, which results in more auxotonic contractions, similar to the situation in vivo. 

PCLS have been shown to have particular importance in lung research due to integrity of the tissue and maintenance of natural cell populations including structural cells (such as epithelial, endothelial, lymphatic, smooth muscle, and fibroblastic cells) [30] and different immune cells (like macrophages, neutrophils, dendritic cells, T cells, and B cells) [31]. Most of these cells are involved in the release of inflammatory cytokines [32,33,34]. Although there is no circulation and infiltration of attracted immune cells, the resident immune cells allow the characterization of immune responses in PCLS [35,36]. These cells are viable and interact reflecting the typical specialized lung functions [37] and local tissue responses [32]. In the field of veterinary medicine, PCLS from avian [38], equine [39], swine [40], caprine [41] including bovine [42,43] species have been used in recent years, mainly to study viral infections. 

The aim of this study was to establish ex vivo infection models for studying host-pathogen interactions for caprine and bovine mycoplasma using PCLS, which mimic the in vivo situation in a cost-effective, fast and reproducible manner. For this, we first developed a PCLS model using caprine and bovine lung tissues. Then, we standardized the two infection models for both subspecies of *M. mycoides*, particularly *M. mycoides* subsp. *capri* (*Mmc*) strain GM12 and *M. mycoides* subsp. *mycoides* (*Mmm*) strain Afadé. Using different experimental approaches, we aimed to mimic (I) the initial steps of the infection, namely adherence and the subsequent colonization of the tissue by the mycoplasma and (II) an advanced (acute) stage of infection, where the pathogens are present in high numbers within the host tissue. We evaluated the tissue colonization and pathomorphological changes over time. Results were compared with lungs from experimentally infected goats and cattle.

## 2. Results

### 2.1. Viability and Metabolic Activity of Bovine and Caprine PCLS

The viability, metabolic activity and structural stability of PCLS in culture were monitored over two weeks by different methods. Ciliary activity was observed daily using light microscopy (Figure S1A). Only slices with full ciliary activity (Figure S1A and Supplementary video 1) were used for infection experiments. Uninfected slices showed full ciliary activity for at least two weeks. Furthermore, metabolic activity was determined using a cell proliferation assay. As shown in Figure S1B, slices showed full metabolic activity for up to two weeks.

Structural stability of the lung tissue was confirmed by H&E and IF staining of thin sections from PCLS at different time points. The characteristic structures of the lung parenchyma and airways remained unchanged throughout the duration of the experiment (Figure S1C–H). Overall, no significant changes in viability and structural stability of the tissue slices were observed. 

### 2.2. Adherence of M. mycoides to Caprine PCLS

Adherence and colonization of *M. mycoides* in PCLS was studied by infecting the slices with *Mmc* GM12 and *Mmm* Afadé for four hours and removing unbound bacteria at four hours post infection (hpi). PCLS were incubated for up to five days with washing and change of medium every 24 h. 

**Figure 1:**
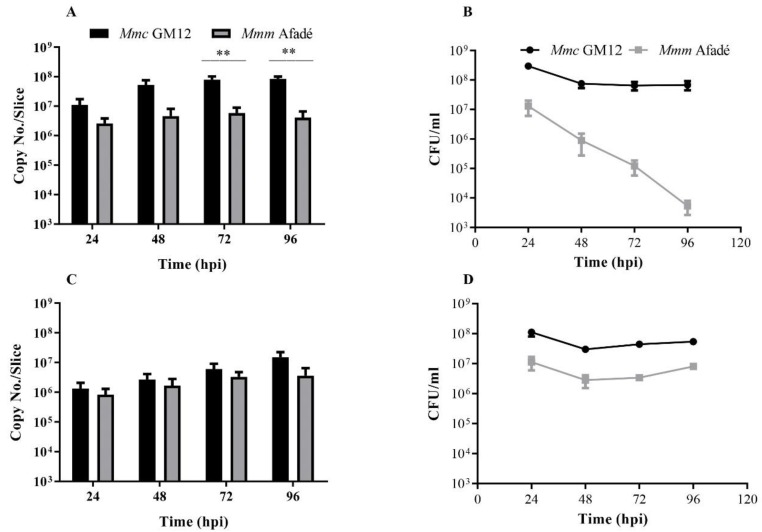
*Mycoplasma mycoides* titer in caprine and bovine precision cut lung slices (PCLS). Adherent bacteria to caprine (**A**) and bovine (**C**) PCLS were determined using qRT-PCR, while the number of non-adherent bacteria released to the media in caprine (**B**) and bovine (**D**) PCLS were determined via plating serial dilutions of culture supernatants on PH agar plates. Adherent *Mmc* GM12 increased in caprine PCLS (**A**) in time, however, a significant decrease in the number of non-adherent *Mmm* Afadé was observed in caprine PCLS (**B**). In bovine PCLS, adherent *Mmc* GM12 and *Mmm* Afadé showed an increase in number in a time-dependent manner (**C**) and the non-adherent bacteria remained constant throughout the infection period (**D**). Three biological replicates, error bars indicate SEM, **p-value < 0.01.

Adherent *Mmc* GM12 cell numbers increased in a time-dependent manner, with a strong increase between 24 and 48 hpi, followed by a slight increase up to 96 hpi (Figure 1A).

Since the PCLS were thoroughly washed and supplied with fresh medium every 24 h, the non-adherent bacteria found in the medium at the end of each 24 h cycle are indicators of viable adherent bacteria in the PCLS. Thus, culture supernatants were used for quantifying the replication efficiency of adherent bacteria by plating serial dilutions on PH agar plates at similar time points as above. We observed that the number of non-adherent *Mmc* GM12 in caprine PCLS remained constant throughout the infection period (Figure 1B). This indicates that *Mmc* GM12 released from the PCLS into the supernatant remained constant over time. 

In addition to *Mmc* GM12, caprine slices were also infected with the same number of viable *Mmm* Afadé. Adherence and colonization were observed, and results compared with *Mmc* GM12 infected caprine PCLS samples. Under similar conditions, the number of adherent *Mmm* Afadé in caprine PCLS at 24 hpi was lower than *Mmc* GM12 and increased only slightly up to 72 hpi. After 72 h, the number of adherent *Mmm* Afadé declined. Throughout the infection period, the number of adherent *Mmm* Afadé remained significantly lower compared to *Mmc* GM12 (p-value < 0.01) (Figure 1A). 

There was a strong decrease in the number of non-adherent *Mmm* Afadé in the supernatant of caprine PCLS (Figure 1B), reflecting a decreasing number of adherent viable *Mmm* Afadé in caprine PCLS. This decrease was less pronounced for tissue adhering *Mmm* Afadé (Figure 1A). 

### 2.3. Adherence of M. mycoides to Bovine PCLS

The adherence of *Mmm* Afadé to bovine PCLS was investigated by the same experimental setup as described above for the caprine PCLS. The number of adherent *Mmm* Afadé in the bovine PCLS showed a consistent increase over time, as shown by qRT-PCR analysis (Figure 1C). Moreover, the number of non-adherent *Mmm* Afadé, which was released into the medium, was also found to be almost constant throughout the infection period (Figure 1D). 

In addition, *Mmc* GM12 was used to infect bovine PCLS. The number of *Mmc* GM12 adhering to the bovine PCLS increased in a time-dependent manner, and the number of non-adherent *Mmc* GM12 remained constant in the bovine PCLS (Figure 1C,D, respectively).

As determined by qRT-PCR with bovine and caprine CEACAM18 genes, the number of cells in both caprine and bovine PCLS were comparable and highly reproducible (Figure S2A,B)

### 2.4. Colonization and Tissue Tropism of Mycoplasma mycoides

The pattern of PCLS colonization by both *Mycoplasma* subspecies was analyzed by immunofluorescence (IF) and immunohistochemistry (IHC). 

**Figure 2:**
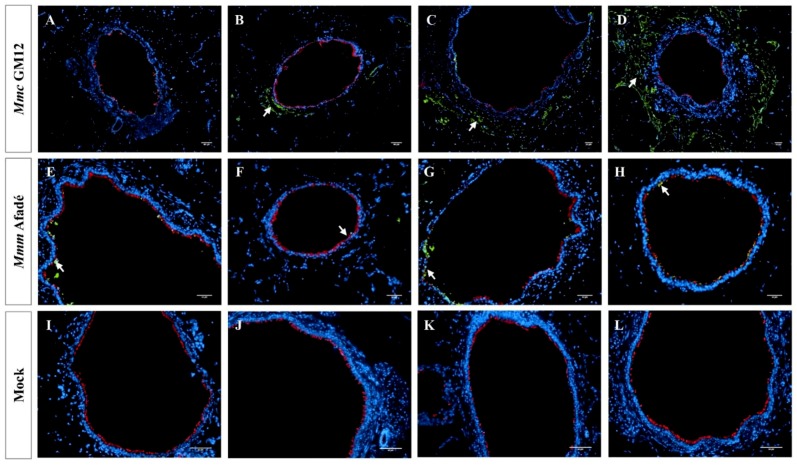
*Mycoplasma mycoides* infection of caprine PCLS Caprine PCLS infected with *Mmc* GM12 (**A**–**D**), *Mmm* Afadé (**E**–**H**), and uninfected control (**I**–**L**). Slices were fixed after 24 h **(A**,**E**,**I)**, 48 h (**B**,**F**,**J**), 72 h **(C**,**G**,**K)**, and 96 h (**D**,**H**,**L**) p.i. *Mmc* GM12 colonizes the sub-bronchiolar tissue, and the invasion increased over time (**A**–**D**, white arrows). *Mmm* Afadé was mainly seen on the ciliated epithelial cells (**E**–**H**, white arrows). This indicates the difference in the tropism of both strains. There were no Mycoplasma cells in the uninfected control samples (**I**–**L**). Immunofluorescence images of tissue sections are shown, labeled with a polyclonal rabbit anti-*Mmm* PG1 antibody combined with a FITC-labeled goat anti-rabbit IgG secondary antibody (green) and a mouse monoclonal anti-β-tubulin-Cy3 antibody (red). Nuclei of caprine cells were counterstained with DAPI (blue). Scale bars: 50 µm.

**Figure 3:**
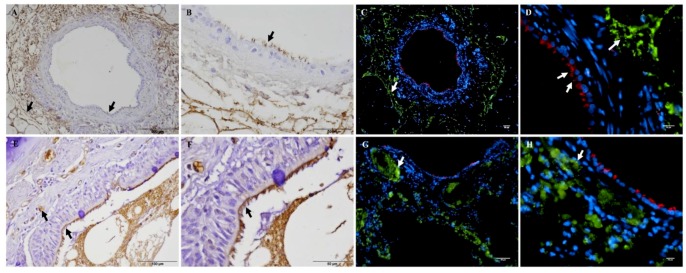
Comparison of sub-bronchiolar distribution and cell tropism of *Mmc* GM12 in caprine PCLS and lungs of experimentally infected goats. Sub-bronchiolar distribution of *Mmc* GM12 in caprine PCLS, 96 hpi (**A–D**) and goats infected with *Mmc* GM12 (**E**–**H**). IHC of caprine PCLS 96 hpi showed a high amount of *Mmc* GM12 in the sub-bronchiolar tissue (**A**, black arrows) and a close-up view of the same sample (**B**, black arrows), revealing complete coverage of ciliated cells with *Mmc* GM12. Seriate sections were stained with IF and were comparable to the IHC analysis (**C**,**D**, white arrows). Comparison of PCLS with lungs of experimentally infected goats showed similar results in IHC (**E**,**F**, black arrows) and IF (**G**,**H**, white arrows). Red: β-tubulin of ciliated cells, Green: *Mmc*, Blue: Nuclei of caprine cells. Scale bars: A = 200 µm, B, C, F and G = 50 µm, D and H = 10 µm, E = 100 µm.

In infected caprine PCLS, both strains adhered to ciliated epithelial cells (Figure 2). However, we observed striking differences in tissue colonization. *Mmc* GM12 was found in the lamina propria, muscularis mucosa extending to the tunica adventitia and further to the alveolar epithelial cells, which we referred to this area as sub-bronchiolar or sub-bronchial space. The colonized area increased over time (Figure 2A–D) and closely resembled the distribution of *Mmc* GM12 in the lungs of goats experimentally infected with *Mmc* GM12 (Figure 3A–D in PCLS versus E–H in vivo). Both IF and IHC stainings revealed similar results in PCLS and in lung samples from experimentally infected goats. Interestingly, using IHC, we detected *Mmc* GM12 adherent to the cilia of almost all ciliated epithelial cells in PCLS (Figure 3A,B, black arrows) and considerable area of ciliated epithelial cells in goat lungs experimentally infected with *Mmc* GM12 were also covered with the bacterium (Figure 3E,F, black arrows). On the other hand, *Mmm* Afadé adhered mainly to ciliated epithelial cells (Figure 2E–H). 

**Figure 4:**
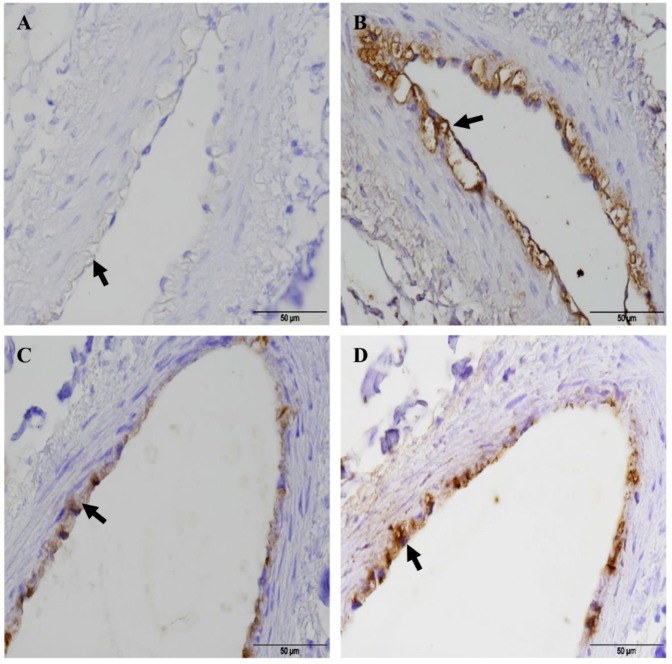
Adherence of *Mmc* GM12 to caprine and bovine pulmonary endothelial cells. IHC showing endothelial cells stained with anti-Von Willebrand Factor antibody (a marker of endothelial cells) in caprine (**A**, black arrow) and bovine (**C**, black arrow) PCLS. Seriate sections were stained with anti-*Mmm* PG1 antibody to see adherent *Mmc* GM12 to the caprine (**B**, 96 hpi, black arrow) and bovine (**D**, 48 hpi, black arrow) endothelial cells in PCLS. Red- β-tubulin of ciliated cells, Green: *Mmc*, Blue: Nuclei of caprine cells. Scale bars = 50 µm.

*Mmc* GM12 was detected in the “paracellular space” of the bronchiolar epithelium (Figure S3) and on endothelial cells (Figure 4). With regards to the attachment to the endothelial cells, seriate sections were taken to stain blood vessels and *Mmc* GM12. Anti-Von Willebrand factor antibody was used as a blood vessel marker for IHC. Accordingly, *Mmc* GM12 was found adherent to caprine (Figure 4A,B) and bovine endothelial cells (Figure 4C,D), which is in line with the invasive properties of strain GM12 causing septicemia. 

**Figure 5:**
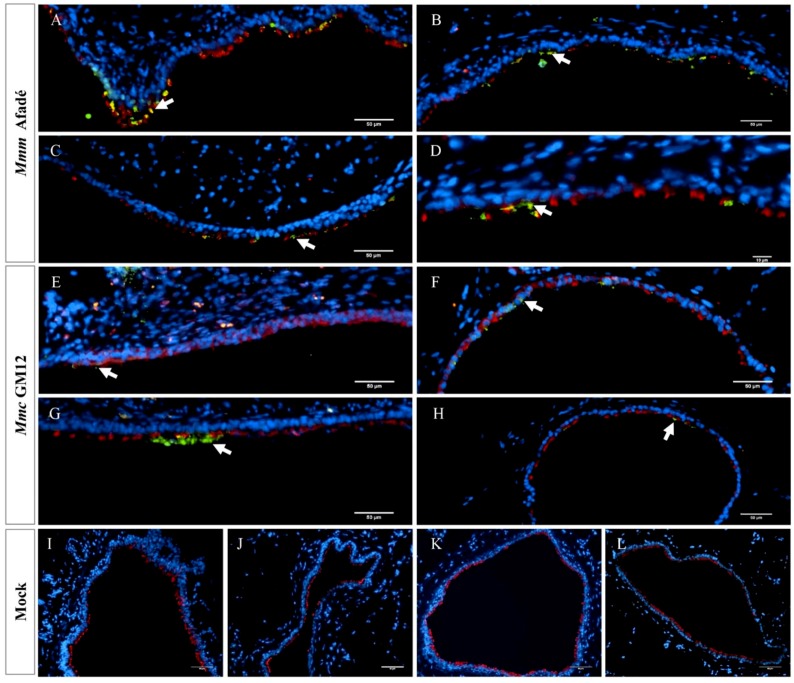
*Mycoplasma mycoides* infection of bovine PCLS. Bovine PCLS infected with *Mmm* Afadé (**A**–**D**), *Mmc* GM12 (**E**–**H**) and uninfected control (**I**–**L**). Slices were fixed after 24 (**A**,**E**,**I**), 48 (**B**,**F**,**J**), 72 (**C**,**G**,**K**) and 96 (**D**,**H**,**L**) hpi. Both strains showed higher tropism to the ciliated epithelial cells. There were no Mycoplasma cells in the uninfected control samples (**I**–**L**). Red: β-tubulin of ciliated cells, Green: *Mmm* Afadé (**A**–**D**) or *Mmc* GM12 (**E**–**H**), Blue: Nuclei of caprine cells. Scale bars: 50 µm.

Similar to caprine PCLS, in bovine PCLS we observed high amounts of *Mmm* Afadé (Figure 5A–D, Figure S4) as well as *Mmc* GM12 (Figure 5E–H) cells adherent to the ciliated epithelial cells. Only occasionally, *Mmm* Afadé was observed in the alveolar tissues (data not shown). The uninfected controls remained free from mycoplasma (Figure 4I–L).

**Figure 6:**
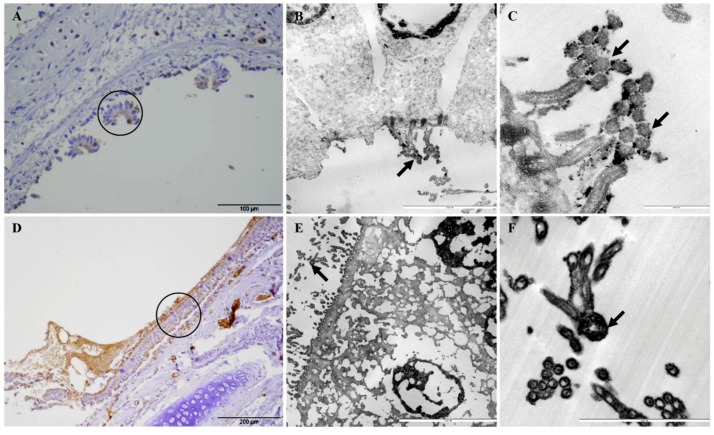
Electron microscopic analysis of *Mmc* GM12 adherence to ciliated cells. The regions of IHC stained caprine PCLS infected with *Mmc* GM12 (**A**, black circle) and infected goat lung (**C**, black circle) were used for electron microscopy. Both samples revealed adherence of *Mmc* GM12 to the ciliated cells (**B**,**C**,**E**,**F**, arrows). Scale bars: A = 100 µm, B = 5 µm, C = 1 µm, D = 200 µm, E = 10 µm, F = 2.5 µm.

**Figure 7:**
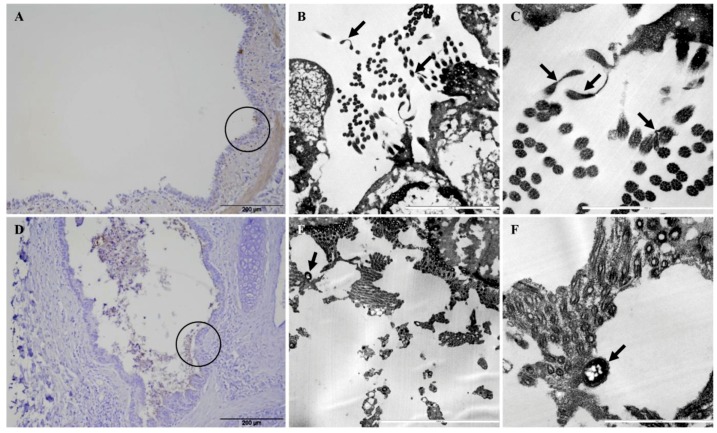
Electron microscopic analysis of *Mmm* Afadé adherence to ciliated cells. The regions of IHC stained bovine PCLS infected with *Mmm* Afadé (**A**, black circle) and infected cattle lung (**D**, black circle) were used for electron microscopy. Both samples revealed adherence of *Mmm* Afadé to the ciliated cells (**B**,**C**,**E**,**F**, arrows). Scale bars: A and D = 200 µm, B = 5 µm, C and F = 2.5 µm, E = 1 µm.

Adherence of *Mmc* GM12 and *Mmm* Afadé to the ciliated (bovine and caprine, respectively) bronchiolar (bronchial) epithelial cells was further investigated using electron microscopy of infected PCLS and lungs of experimentally infected animals (both caprine and bovine). Samples were taken from IHC stained sections of caprine PCLS and infected goat lung tissue (Figure 6A,D, respectively) as well as bovine PCLS and infected cattle lung tissue (Figure 7A,D, respectively). Results observed in infected PCLS and experimentally infected animals were similar, where *Mmc* GM12 were adherent to the cilia in caprine PCLS (Figure 6B,C) and infected goat lungs (Figure 6E,F). Similarly, *Mmm* Afadé was observed adherent to the cilia of bovine PCLS (Figure 7B,C) and infected cattle lungs (Figure 7E,F). 

### 2.5. Histopathological Changes in Long-Term Infected PCLS Resemble the in Vivo Situation

**Figure 8:**
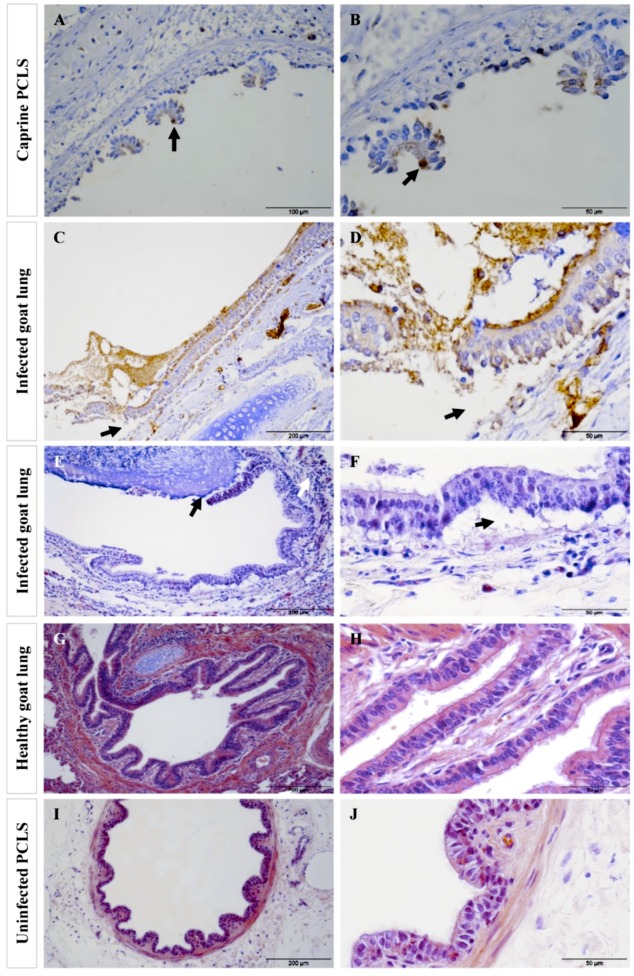
Tissue destruction in caprine PCLS following infection with *Mmc* GM12 in PCLS and lungs of goats infected with *Mmc* GM12 in vivo. After 24 h of continuous infection, extensive detachment and destruction of the bronchiolar epithelial layer were observed in caprine PCLS infected with *Mmc* GM12 (**A**,**B**, IHC staining). Bacteria were mainly adherent to the ciliated cells (**A**,**B**, black arrows) and detach the ciliated cells 24 hpi leaving the basal cells. Similar histopathological changes were found in tissue sections of goat lungs experimentally infected with *Mmc* GM12 (**C**,**D**, IHC staining, **E**,**F**, H&E staining). Both the IHC overview (**C**, black arrows) and close up (**D**, black arrows) and H&E staining overview (**E**, black arrows) and close up (**F**, black arrows) show areas of detachment of the bronchiolar epithelial layer from the basement membrane region. In the in vivo samples, bacteria were highly adherent to the ciliated cells as observed by IHC staining of infected goat lungs (**C**,**D**). Infiltration of leucocytes was also observed in infected samples (**E**, white arrows). Histological section of an apparently healthy goat lung (**G**,**H**) and uninfected PCLS (**I**,**J**) revealed an intact bronchiolar tissue architecture. Scale bars: A = 100 µm, B, D, F, H, and J = 50 µm, C, E, G, and I = 200 µm.

**Figure 9:**
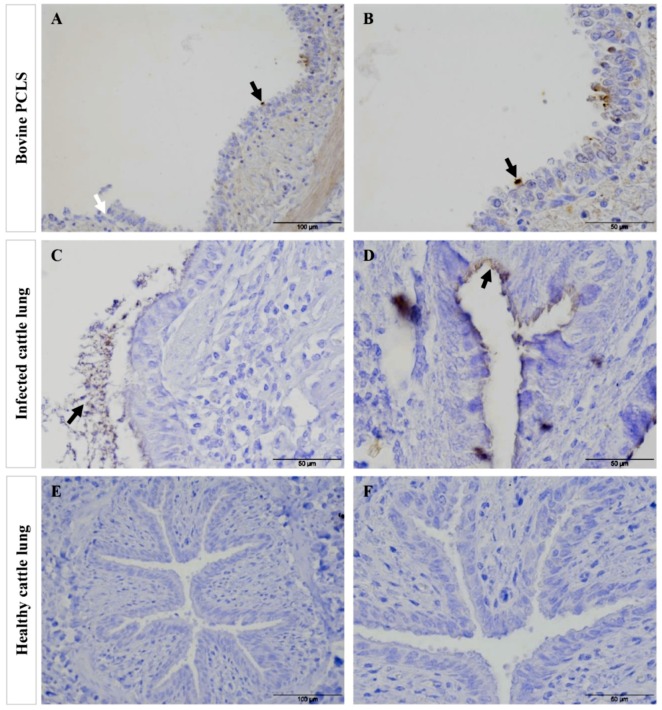
Comparison of tissue destruction of bovine PCLS infected with *Mmm* Afadé versus lungs of cattle experimentally infected with *Mmm* Afadé (IHC). Bovine PCLS infected with *Mmm* Afadé (**A**,**B**), for continuous 24 h. Lungs of cattle infected with *Mmm* Afadé (**C**,**D**). Lung from healthy uninfected cattle (**E**,**F**). Bacteria were mainly adherent to the ciliated epithelial cells both ex vivo in PCLS (**A**,**B**, black arrows) and in vivo (**C**,**D**, black arrows). Destruction of the ciliated epithelial layer was observed in PCLS accompanied by detachment of the epithelial layer (A, white arrows) and partly in cattle infected with *Mmm* Afadé (**C**). Lungs from healthy cattle were also stained similarly and no mycoplasma was detected and revealed an intact bronchiolar tissue architecture (**E**,**F**). Scale bars: A and E = 100 µm, B, C, D, and F = 50 µm.

In a second experimental approach, we infected caprine and bovine PCLS continuously for 4, 8, 12 and 24 h without washing to mimic an advanced (acute) stage of infection, where the pathogens are present in high numbers within the host tissue. The most striking effect we observed after 24 h of continuous infection was massive destruction of the ciliated bronchiolar epithelial cell layer, which was widely detached from the underlying basement membrane. This type of tissue injury was seen in caprine (Figure 8A,B) and bovine (Figure 9A,B) PCLS infected with *Mmc* GM12 and *Mmm* Afadé, respectively. Results from PCLS damage were compared with the tissue damage observed in vivo in goats infected with *Mmc* GM12 (Figure 8C–F) and cattle infected with *Mmm* Afadé (Figure 9C,D). Similar histopathological changes as observed in PCLS were also detected in these in vivo samples. Destruction of the bronchiolar tissue, with the detachment of bronchiolar epithelial cell layer from the underlying lamina propria was also observed in infected goat (Figure 8E,F) and cattle lungs (Figure 9C) similar to our observations in PCLS (Figure 8A,B in goats versus Figure 9A,B). 

Histopathological changes due to experimental infection in vivo and ex vivo infection using PCLS were comparable. Samples from goats experimentally infected with *Mmc* GM12 showed high infiltration of leucocytes (Figure 8E, white arrow) in the lung parenchyma. Lung samples from healthy goats (Figure 8G,H) and cattle (Figure 9E,F) were included and stained in the same way. We never observed any tissue damage in these samples indicating that the tissue damage is associated with the presence of the pathogens but not due to sample processing. 

To examine the development of this tissue damage in more detail, we compared caprine and bovine PCLS continuously infected for 4, 8, and 24 h with *Mmc* GM12 and *Mmm* Afadé, respectively. Using this method, we confirmed that tissue destruction increased in a time-dependent manner, whereby the amount of bacteria on the ciliated cells increased as the time of incubation without washing increased leading to the loss of the upper part of the ciliated epithelium at the end of the experiment (Figure S5A–C for caprine versus E–G for bovine PCLS). Bacteria adherent to the ciliated cells was comparable to the observation in vivo in infected goats (Figure 8C,D) and cattle (Figure 9C,D). In addition, the beginning of ciliostasis was observed after 8–12 hpi in both types of infected PCLS. After 24 hpi, ciliary activity was completely abolished, confirming the association of tissue damage with infection. Bacterial titer in the cell culture medium remained constant during these time points (Data not shown).

Results in both species were compared with uninfected control samples taken at 24 hpi in caprine (Figure S5D) and bovine (Figure S5H) PCLS, where no tissue destruction was observed. Ciliary activity and tissue architecture of the uninfected controls were unchanged throughout the duration of the experiment. 

Bovine PCLS were also infected with two additional virulent *Mmm* strains, strain Gladysdale (Figure S6A,B) and strain B237 (Figure S6C,D) and we observed the same type of tissue destruction using H&E and IHC staining. Both strains also adhered to the ciliated epithelial cells, as observed by IHC (Figure S6B,D). 

**Figure 10:**
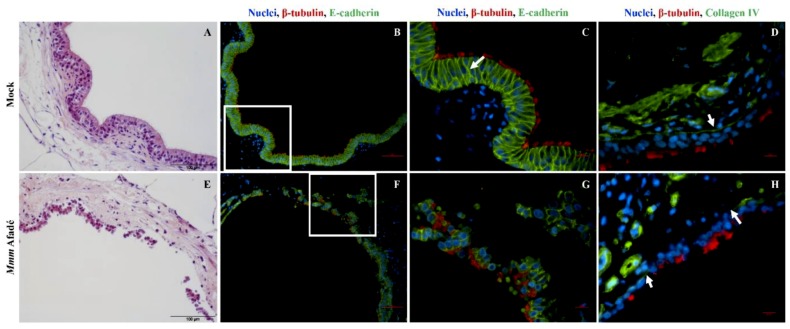
Effects of 24 h infection with *Mmm* Afadé on the epithelial barrier in bovine PCLS. In uninfected control slices, the integrity of the epithelial barrier is demonstrated by H&E (**A**) and Immunofluorescence (**B**–**D**) staining of PCLS sections. Ciliated epithelial cells are connected by E-cadherin (**B**,**C**, white arrow) and the epithelial cell layer is attached on the Collagen IV containing basement membrane (**D**, white arrow). After 24 h of continuous infection with *Mmm* Afadé, the epithelial cell layer is detached from the sub-bronchiolar tissue (**E**), the connections between ciliated epithelial cells are partly broken (**F**,**G**) and the Collagen IV layer is largely degraded (**H**, white arrows). C and G are close-up views of the white rectangles on B and F, respectively. For IF images, PCLS sections were labeled with a monoclonal mouse anti-E-cadherin antibody or a polyclonal rabbit anti-collagen type IV antibody, combined with corresponding Alexa fluor 488 labeled secondary antibodies. Cilia (red) and nuclei (blue). Scale bars: H&E stains = 100 µm, B and F = 50 µm, D and H = 10 µm.

We investigated the area of the detached epithelial layer in more detail by IF staining of the adherens and tight junction proteins including E-cadherin, β-catenin, and occludin. In addition, Collagen IV, which is a key component of the basement membrane that separates epithelial cells from the underlying lamina propria was also investigated (Figure 10 and Figure S7). Accordingly, in uninfected bovine PCLS, E-cadherin and collagen IV stains confirmed the typical architecture of the bronchiolar epithelial barrier, the tight epithelial cell-to-cell adherence and a continuous basement membrane (Figure 10A–D). At 4 hpi, tissue architecture remained unchanged. However, we found out that PCLS after continuous 24 h infection lost epithelial integrity, as evidenced by H&E and IF stainings (Figure 10E–G). In addition, we also observed damage of collagen IV in infected PCLS samples (Figure 10H and Figure S7). The detached sheets of bronchiolar epithelial cells found in *Mmm* Afadé infected bovine PCLS consisted of groups of epithelial cells still connected by adherens junctions and covered with cilia on their apical side (Figure 10F,G and Figure S7). 

## 3. Discussion

Mycoplasma-related ruminant diseases did not attract as much scientific attention as other livestock diseases, despite the fact that they have an enormous economic impact. One reason for this is the biosecurity level that applies for pathogens such as *Mmm* and *M. capricolum* subsp. *Capripneumoniae*, another is the lack of small animal models. Infections of cattle and goats are time-consuming and expensive, and robust reproducible challenge models are not always in place [19]. Thus, the research on mycoplasma would greatly benefit from infection models that mimic the in vivo situation. Clearly, ex vivo models are the closest approximation to the in vivo situation, and such systems outcompete *in vitro* cell culture, which is a relatively easy to standardize system but an oversimplification of the complex host target tissue, especially in the case of mycoplasma that affects the respiratory system. The proteome of pleural effusion during CBPP has been recently characterized by our group [44] revealing the presence of mycoplasma proteins in vivo that are subject to ongoing studies.

In the present study, we explored the capacity of bovine and caprine precision cut lung slices (PCLS) as ex vivo infection models to study host-pathogen-interaction of ruminant mycoplasma. For this, we selected the closely related pathogens *Mmm* and *Mmc*, which infect cattle and goats, respectively. Despite the high genetic similarity between *Mmm* and *Mmc*, the two subspecies differ greatly in terms of host susceptibility and pathogenesis [45,46]. Only a few reports describe the isolation of *Mmm* in small ruminants, particularly in goats [47,48,49]. There are also some reports on the isolation of *Mmc* from cattle, but the causal relationship between *Mmc* isolation and the observed disease patterns such as abortion [50] or CBPP like disease [51], are yet unproven. Although distinct differences in host and tissue tropism between *Mmm* and *Mmc* are well known, the underlying mechanisms are poorly characterized.

The PCLS set up in this study remained reproducibly viable over a minimum of two weeks, as shown by ciliary and metabolic activity assays. This was in agreement with Temann and colleagues [32], who demonstrated that the kinetics of metabolic activity in PCLS remained constant for up to 14 days. In our initial experimental setup, we removed unbound bacteria 4 hpi and analyzed the temporal development of adherence and bacterial distribution in both caprine and bovine PCLS up to four days p.i. Ciliary activity is one of the control parameters in studying the pathogenesis of viruses or bacteria that attack the airway system. Certain strains of swine influenza viruses have been reported to be ciliostatic and this has been confirmed using PCLS [40]. Ciliostasis has been reported in mycoplasma infections [52] and was also proven using porcine, mouse, and chicken tracheal organ cultures [52,53,54]. In our current study, *Mmm* Afadé and *Mmc* GM12 did not influence ciliary activity in our first infection model, where unbound bacteria were removed after 4 hpi and subsequent incubation for four days with washing and medium change every 24 h. However, ciliostasis in both species with both mycoplasma strains was observed after a minimum of eight hours continuous infection of PCLS (without removing the unbound bacteria at 4 hpi as in our first experimental setup). This indicates that in the first infection protocol, the bacterial titer and/or the concentration of bacterial metabolites are probably too low to induce ciliostasis. On the other hand, if the pathogen titer is kept constantly high, ciliostasis and tissue destruction were observed. With these, we could conclude that we are able to mimic the early and advanced or acute phases of infection using these different infection schemes. 

During infection of caprine PCLS with *Mmm* Afadé, we observed a decrease in the number of non-adherent bacteria over time, whereas the non-adherent *Mmm* Afadé titer was slightly increased in bovine PCLS. The number adherent *Mmm* Afadé in caprine PCLS remained constant as revealed by qRT-PCR. However, it should be considered that DNA from dead bacterial cells might have influenced the PCR based quantification of adherent bacteria. The strong reduction in the number of non-adherent *Mmm* Afadé suggests that survival and/or proliferation of *Mmm* Afadé might be host specific. Our result is in agreement with a previous report that *Mmm* is both species and tissue-specific. Aye and colleagues [55] could show significantly higher numbers of *Mmm* adherent to adult primary lung epithelial cells compared to primary caprine lung epithelial cells. On the contrary, the adherence of fast-growing *Mmc* was not strongly influenced by the type of host tissue since we observed a continuous increase in caprine as well as bovine PCLS. 

Both caprine and bovine PCLS infection with *Mmc* GM12 resulted in a comparably higher bacterial number than *Mmm* Afadé throughout the infection period, which we think might be attributed to the better growth of this organism and its less fastidious nature. *Mmc* GM12 in RPMI-2 has shorter (5.8 h) generation time compared to *Mmm* Afadé (11.6 h) (Figure S8), which could result in the increased number of bacteria for a similar infection period. The growths of both *Mmc* GM12 and *Mmm* Afadé in media that we used for our current study were determined and revealed that both subsp. do not grow in the cell culture medium without the addition of serum (Figure S9). 

The analysis of adherence and tissue colonization by IF also revealed marked differences between the two subspecies. It has been reported for different respiratory pathogens, that the ciliated epithelial cells are the major targets for adhesion [42,43]. Our study revealed that both *Mmm* Afadé and *Mmc* GM12 also adhered to the ciliated epithelial cells in both caprine (Figure 2 and Figure 3) and bovine (Figure 5 and Figure S4) PCLS, which is comparable to the result observed in vivo in goats infected with *Mmc* GM12 (Figure 3 and Figure 8) and cattle infected with *Mmm* Afadé (Figure 9C,D). 

As reported by Aye et al. [55], *Mmm* is less adherent to endothelial cells than to epithelial cells. Similarly, we did not detect *Mmm* Afadé adherent to endothelial cells of the blood vessels present in the PCLS and from cattle infected with *Mmm* Afadé (Data not shown). However, Di Teodoro and colleagues [56] reported *Mmm* strongly adherent to endothelial cells. The different observations might be due to different experimental systems, tissue composition (in respiratory explants, trachea, bronchus, and lung parenchyma were separately used for the study, whereas PCLS includes bronchioles and lung parenchyma together), culture conditions, initial duration of infection (1 h vs. 4 h), thickness of tissue samples (1mm vs. 0.3 mm), preparation of samples and reproducibility of tissue size (manual sections vs. automated slicer), and media composition. In addition, *Mmm* Afadé is reported to undergo intrastrain phase variation, associated with differential capsule-production resulting in an opaque (OP, capsulated variant) and translucent (TR, non-capsulated variant) colony types [57]. It has been shown that the resistance to innate immunity, adaptation to host tissues and microniches, adherence to abiotic surfaces, and others vary between these two colony types [58]. Further investigation should be conducted to find out the abundant colony variant of *Mmm* Afadé in the PCLS infection model. Our IF staining of caprine PCLS infected with *Mmc* GM12, on the other hand, clearly showed adherence of *Mmc* GM12 to caprine (Figure 4B) and bovine (Figure 4D) pulmonary endothelial cells. Despite the high homologies between these two subspecies, this difference could also explain the invasive nature of *Mmc* GM12. In general, from our adherence and quantification studies, we conclude that *Mmm* Afadé shows host (species) specificity, whereas *Mmc* GM12 can infect tissues from both host species.

An additional difference we observed was the invasion of *Mmc* GM12 into the sub-bronchiolar tissue in infected caprine PCLS (Figure 2B–D and Figure 3A–D), which is in accordance to its septicemic nature. Even though PCLS have a “cut surface”, which is in contact with the bacterial suspension during infection, it seems unlikely that the observed sub-bronchiolar colonization starts from there. We stained thin sections prepared from the inner part of the PCLS, thereby excluding bacteria, which adhere randomly on the cut surface. Within the thin sections, *Mmc* GM12 is not found equally distributed but accumulated around the bronchioles, and the bacteria were also detected in the paracellular space of ciliated epithelial cells (Figure S3). This seems to be a specific host-pathogen interaction since a similar distribution of *Mmc* GM12 and *Mmm* Afadé in bovine PCLS was not detected. In summary, we assume that *Mmc* GM12 enters the PCLS via the bronchioles, crosses the epithelial cell layer via the paracellular space and subsequently colonizes the sub-bronchiolar tissue. Paracellular movement as an entry mechanism to the subepithelial tissue was reported for other invasive pathogens such as *Streptococcus pneumoniae* and *Haemophilus influenza* [59,60].

The distribution of *Mmc* GM12 observed in caprine PCLS is highly similar to the one found in the lungs of experimentally infected goats. The observed differences could reflect the more systemic nature of infections with *Mmc* GM12. This strain was originally isolated from septicemic children [61] and our findings support its ability to spread across the epithelial barrier, to adhere as well as replicate in endothelial cells, thereby disseminating throughout the body, resulting in MAKePS [13]. Further investigations are needed to analyze the mechanism, how *Mmc* crosses the ciliated epithelial layer and in/on which cell types in the sub-bronchiolar tissue it resides.

For mimicking an advanced stage of infection, PCLS were infected for 24 h continuously (without removing the unbound bacteria at 4 hpi as in our first experimental setup). Under these conditions, the histopathological changes observed in caprine and bovine PCLS were comparable to those seen in the lungs of experimentally infected, septicemic goats and cattle. Similar histopathological observations were reported in *Mmc* GM12 infected goats [62]. To analyze the observed tissue damage, we tested the integrity of adherens and tight junctions of the bronchiolar epithelial cells using E-cadherin, β-catenin and occludin staining. We found that adherens junctions were partially destroyed and groups of bronchiolar epithelial cells were detached from the basement membrane. The basement membrane itself was destroyed in areas of epithelial cell detachment, as judged by collagen IV staining (Figure 8 and Figure S7). Based on our 24 h infection, we hypothesize that during long-term infection, both *Mmm* and *Mmc* can break the adherence and tight junctions, destroy the basement membrane thereby breaching the inter-epithelial cells and translocate into the underlying sub-bronchiolar tissue, which has been shown to be scattered in lung tissues derived from CBPP lesions [63]. The disruption of the basement membrane during infectious diseases often involves proteases [64]. These enzymes can be either produced by the pathogenic bacteria themselves or the pathogens might indirectly activate or modify proteolytic enzymes of the host [65,66]. Notably, mycoplasma has a very small genome and toxins, invasins or other direct virulence-associated factors have not yet been identified. Thus, metabolic activities and specific cell surface components contribute to their pathogenicity and survival in their host [57,67]. Production and translocation of reactive oxygen species such as Hydrogen peroxide (H_2_O_2_) could also result in the destruction of host cells [67,68]. The detailed mechanism of tissue destruction by *Mmm* and *Mmc* as observed in our study remains to be elucidated by further investigations. 

In conclusion, PCLS represent a suitable ex-vivo model for studying ruminant mycoplasma that infects the respiratory system. Using this model, we were able to show host specificity of *Mmm*. Interestingly, *Mmc* GM12 showed a particular high adherence to the sub-bronchiolar or basal epithelial cells and alveolar tissue. Higher adherence of *Mmc* GM12 was also observed in the endothelial cells, which is in line with the systemic nature of this strain. Using PCLS, we were able to mimic acute phases of infection caused by *Mmc* GM12 and *Mmm* Afadé in goats and cattle, respectively. We confirmed that pathological changes in PCLS after infection with *Mmc* GM12 resemble those seen during infection of goats. We speculate that *Mmc* GM12 can pass the epithelial cells by destroying the tight and adherens junctions. In contrast, even though *Mmm* Afadé destroys these junctions, it remains attached to the ciliated epithelium. Future studies involving transcriptomic and immunologic analysis are required to identify factors, which will help to dissect the underlying mechanisms of tropism and tissue damage. This might contribute to the identification of candidates for better diagnostics, treatment, and/or immune prophylaxis of mycoplasma infections in livestock.

## 4. Materials and Methods

### 4.1. Chemicals, Bacterial Strains and Culture Conditions

All chemicals used in this study were purchased from Carl Roth (Karlsruhe, Germany), unless stated otherwise. Bacterial strains used in this study are *Mycoplasma mycoides* subsp. *capri* (*Mmc*) strain GM12 [61] and pathogenic isolates of *Mycoplasma mycoides* subsp. *mycoides* (*Mmm*) strains Afadé [69], Gladysdale [70], and B237 [69]. *Mycoplasma* strains were grown in modified PH medium [71] supplemented with 5% heat-inactivated bovine serum (Pan-Biotech, Aidenbach, Germany) for two days at 37 °C. The number of viable mycoplasma cells was determined as colony forming units per milliliter (CFU/mL) by plating serial tenfold dilution on PH agar plates [71,72]. 

### 4.2. Preparation and Maintenance of PCLS 

Bovine and caprine lungs were collected from apparently healthy adult cattle and goats (different breeds) from local slaughterhouses in Germany. Lungs were obtained immediately after slaughter, chilled on ice and transported to the lab and processed within one hour. Control samples were immediately fixed with 4% Parafolmadehyde (PFA) in PBS for 24–48 h. 

During one batch of PCLS preparation, we included lungs from at least 2 animals and results included in this paper are from a minimum of three biological replicates for each host species. Precision cut lung slices were made according to the method described by Kirchhof et al. [41]. Briefly, accessory and cranial lobes of the lungs were removed and filled with 1.5% low melting agarose (GERBU, Heidelberg, Germany) in RPMI 1640 medium (ThermoFischer Scientific, Darmstadt, Germany). The agarose in the lung tissue was allowed to cool for about 30 min on ice. Cylindrical sections were stumped out and sliced using Krumdieck tissue slicer (approx. 300 µm thickness). PCLS with bronchioles were transferred to 24 well plates, washed with prewarmed phosphate buffered saline (PBS) and incubated at 37 °C and 5% CO_2_. Samples were washed and medium changed every day (for three days). The first medium (RPMI-1) contains RPMI1640-Medium with antibiotics and antimycotics (10 mL/L of penicillin–streptomycin stock solution containing 5000 units/mL penicillin and 5000 µg/mL streptomycin, 50 mg/L kanamycin, 2.5 mg/L amphotericin B, and 1 mg/L clotrimazole), (all except amphotericin B were purchased from Sigma-Aldrich, Munich, Germany). After complete removal of the agarose on the 3rd day, PCLS were transferred to RPMI-2, which contains RPMI1640-medium, penicillin (10,000 units/L), amphotericin B (2.5 mg/L), clotrimazole (1mg/L), and 20% heat-inactivated bovine serum (Pan-Biotech, Aidenbach, Germany) to allow the growth of *Mycoplasma* spp. The doubling times (Td) of *Mmc* GM12 and *Mmm* Afadé in RPMI-2 were determined using Picogreen assay according to the method described in [73]. Fluorescence was measured using SpectraMax i3X (Molecular Devices, California, United States) with excitation at 488 nm and detection of emission at 525 nm. 

Lung samples from goats and cattle experimentally infected with *Mmc* GM12 [62] and *Mmm* Afadé [74] were included in this study for comparison with PCLS. Goat and cattle lungs from apparently healthy animals as negative controls were included.

### 4.3. Quality Control of PCLS

The PCLS were checked for ciliary activity, as described by Punyadarsaniya et al. [75]. Briefly, each bronchiole was virtually divided into ten segments. All segments were monitored for the presence or absence of ciliary activity using light microscopy. Slices were selected that showed 100% ciliary activity at the beginning of the experiment. 

Metabolic activity was determined using 5-(3-carboxymethoxyphenyl)-2-(4,5-dimenthylthiazoly)-3-(4-sulfophenyl) tetrazolium inner salt (MTS) assay (CellTiter 96® Aqueous One Solution Cell Proliferation Assay) from Promega according to manufacturer’s instructions.

### 4.4. Infection of PCLS

In the first part of our study, we investigated the dynamics of adherence and tissue distribution of *Mmc* GM12 and *Mmm* Afadé in both caprine and bovine PCLS. For that purpose, we infected the PCLS for four hours using 500 µl freshly grown *Mmm* Afadé or *Mmc* GM12 mixed with 500 µL of RPMI-2 medium (48 h culture, approx. 10^8^ CFU/slice). Unbound bacteria were removed four hours post infection (hpi) by washing twice using sterile PBS and PCLS were further incubated in RPMI-2 media for five consecutive days whereby slices were washed, and medium changed every 24 h. The uninfected controls were incubated in a similar medium composition with the addition of 50% PH medium to the RPMI-2 (Only RPMI-2 was also used for maintaining uninfected control samples. In addition, samples from infected and uninfected controls were washed twice and preserved every 24 hpi for further analysis such as DNA extraction for quantitative real-time PCR (qRT-PCR) and fixed in 4% paraformaldehyde (PFA) in PBS for staining. 

In a second experimental approach, we examined caprine and bovine PCLS infected with 10^8^ CFU/slice of *Mmc* GM12 or *Mmm* Afadé for continuous 24 h without washing at 4 hpi. These conditions were chosen to mimic an advanced (acute) stage of infection, where the pathogens are present in high numbers within the host tissue. We evaluated the histopathological changes in PCLS in comparison with lungs of goats and cattle experimentally infected with *Mmc* GM12 and *Mmm* Afadé, respectively. Lungs from apparently healthy goats and cattle were included as uninfected controls. We included intermediate time points (4, 8, and 24 hpi) to investigate the association of tissue damage with the presence of infection and samples were preserved for histological analysis. During this time of infection, the control samples were incubated in RPMI-2 medium plus 50% of PH medium to have a comparable medium composition with infected samples.

### 4.5. Staining of PCLS 

In addition to the ciliary activity and gross morphological examination of the lungs during PCLS preparation, we also did haematoxylin and eosin (H&E), immunofluorescence (IF) and immunohistochemistry (IHC) stains. PCLS for H&E, IF, and IHC stains were fixed with 4% paraformaldehyde (PFA) in PBS for 24–48 h. After fixation, samples were washed with PBS and embedded in paraffin blocks for sectioning. In addition, tissue from apparently healthy goat and cattle lungs, as well as lung samples from experimentally infected goats and cattle, were used to compare results with PCLS. Thin paraffin sections of 3–4 µm were made using rotary microtome. For staining purposes, samples were deparaffinized and rehydrated. The quality of thin sections was checked using H&E staining following standard procedures. IF staining was performed according to the method described in Kirchhoff et al. [41] with modifications. Briefly, blocking of non-specific sites was done using 5% BSA in PBS. Primary antibody for mycoplasma (polyclonal rabbit anti *Mmm* PG1, 1:250) was used, which was stained using goat anti-rabbit IgG (H+L)-FITC secondary antibody (Jackson ImmunoResearch Laboratories, West Grove, USA Dianova). The β-tubulin of the cilia was stained using a mouse monoclonal anti-β-tubulin−Cy3 antibody (1:500, Sigma-Aldrich, Munich, Germany). Adherens junctions in the bronchiolar epithelial cells were stained using purified monoclonal mouse anti-E-cadherin antibody (1:500, BD Transduction Laboratories Heidelberg, Germany) polyclonal rabbit anti-β-catenin antibodies (1:500, Abcam, Cambridge, UK), and monoclonal mouse anti-occludin Alexa fluor 488 antibody, (1:500, ThermoFischer Scientific, Darmstadt, Germany). Polyclonal rabbit anti-collagen type IV antibody (1:400, OriGene Technologies, Herford, Germany) was used to stain the basement membrane. Blood vessels were stained using polyclonal rabbit anti-human von Willebrand factor antibody (1:3000, DakoCytomation, Denmark). All secondary antibodies were diluted 1:1000 in the blocking buffer. The nuclei were stained using 4’,6-diamidino-2-phenylindole (DAPI), which was incorporated in the ProLong™ Gold Antifade Mountant (ThermoFischer Scientific, Darmstadt, Germany). Visualization of IF stains was made using Nikon Eclipse Ti-S inverted fluorescence microscope. Immunohistochemistry was done using Dako Envision Kit (Agilent, Santa Clara, USA) according to the manufacturer’s protocol. H&E and IHC stained sections were visualized by Zeiss axioskop equipped with Olympus DP 70 digital camera. 

### 4.6. Electron Microscopy

To analyze the localization of *Mycoplasma mycoides* in PCLS and lungs, pop-off technique was used to process the samples for electron microscopy according to the method described by Lehmbecker et al. [76]. Briefly, slides stained with IHC were used and the area of interest was marked. The coverslips were removed by rinsing them in xylene. After removing the coverslips, tissue sections were washed twice with 100% ethanol for 5 min each followed by washing with propylene oxide plus 100% ethanol (1:1) for 2 min. Samples were then coated with propylene oxide plus epoxy resin (1:1) for 20 min. A gelatin capsule filled with epoxy resin was placed on the marked area of interest and allowed to polymerize for 1 h at 35 °C, followed by 1 h at 45 °C and finally overnight at 85 °C. The epoxy resin blocks were removed from the glass slide by dipping them in liquid nitrogen. Finally, ultra-thin sections were made with an approximate thickness of 70 nm. These were mounted on mesh copper slides for visualization with electron microscopy. Transmission electron microscopy was performed using Zeiss electron microscope (EM10A/B).

### 4.7. DNA Extraction and Quantitative Real-Time PCR (qRT-PCR)

PCLS samples for DNA extraction were stored in 20% glycerol at −20 °C until processing. DNA extraction was performed using Qiagen DNeasy blood and tissue kit (Qiagen) according to manufacturer’s instructions. Briefly, one PCLS sample was used per preparation. Samples were cut into small pieces and completely lysed with the tissue lysis buffer including proteinase K overnight in a water bath at 56 °C. RNase treatment was conducted before proceeding to the column purification. After RNA digestion, spin column purification was conducted according to the manufacturer’s instructions. 

For quantification of adherent *Mmm* and *Mmc* to the PCLS, qRT-PCR, based on the bovine carcinoembryonic antigen-related cell adhesion molecule 18 (CEACAM18) and the *M. mycoides* putative adenylate kinase (*adk*) genes were used and extended for the application in goat tissue. Standard curves were made using *Mmm* and *Mmc* genomic DNAs, which were prepared using Qiagen genomic DNA extraction kit with 100/G QIAGEN Genomic-tip. The primers used in this study are listed in Table 1.

The qRT-PCR reactions were performed in a total volume of 20 µL reaction mix containing 10 µL of SyberGreen master mix (Qiagen, Hilden, Germany), 2.5 µL of DNA template, 4 µM forward and reverse primers each (oCC18r-fw and oCC18r-rev for bovine, oCC18z-fw, oCC18z-rev for caprine, oMMS_A0796-fw and oMMS_A0796-rev for *Mmm* and *Mmc*), and 5.9 µL of RNase free water. Samples and controls were run in duplicates on a Mx3005P qPCR system (Stratagene, Agilent Technologies, LaJolla, CA, USA). The thermal cycling profile was as follows: one cycle of denaturation at 95 °C for 20 min (segment 1), forty cycles of: 1 min at 95 °C, 1 min at 55 °C and 40 sec at 72 °C (segment 2), and one cycle at 95 °C for 1 min, 30 sec at 55 °C, and 30 sec at 95 °C (segment 3). Copy numbers of bovine and mycoplasma from the PCLS were determined using the ThermoFischer scientific DNA copy number calculator. 

### 4.8. Statistical Analysis 

Growth curves were analyzed using GraphPad Prism version 5.03 (GraphPad, San Diego, CA, USA). Two-way analysis of variance was used to evaluate *Mmm* Afadé and *Mmc* GM12 adherence at different time points in caprine and bovine PCLS. Statistical significance was evaluated based on Bonferroni post-tests and a p-value < 0.05 was considered as statistically significant. Immunofluorescence images were processed with ImageJ 1.51q software (National Institute of Health, USA).

## Figures and Tables

**Table 1:** Primers used in this study.

Gene	Primer ***	Primer Sequence (5’-3’) ***	Primer Binding Positions		Acc. No.	Host
			Downstream	Upstream		
CEACAM18 *	oCC18r-fw	AGCCAAATCTACATCACCCC	149 to 168	-	XM_024979463	Bovine
	oCC18r-rev	ACCTCTAATGGACACACTTT	-	364 to 345		
CEACAM18 *	oCC18z-fw	AGCCAAATCTACATCGCCCC	406 to 425	-	XM_018063197	Caprine
	oCC18z-rev	ACCTCTAACGGACACACTTT	-	621 to 602		
MMS_A0796 **	oMMS_A0796-fw	AGCTTGTTCTAAAGTTCTTG	273 to 254	-	CP002107	*Mmm* Gladysdale
	oMMS_A0796-rev	CTGGTGATTTAATGAGAAAAG	-	92 to 112		

* CEACAM18- Carcinoembryonic -related cell adhesion molecule 18, ** MMS_A0796- putative adenylate kinase, *** Reference: All primers are from this work.

## Supplementary Materials

The following are available online at https://www.mdpi.com/2076-0817/8/2/82/s1, Figure S1. Quality control of PCLS. Figure S2. Comparison of cell numbers in caprine and bovine PCLS. Figure S3. Paracellular localization of *Mmc* GM12 in caprine PCLS (96 hpi). Figure S4. Adherence of *Mmm* Afadé to bovine ciliated epithelial cells (IHC). Figure S5. Stage of tissue destruction in caprine and bovine PCLS (IHC). Figure S6. Destruction of the bronchiolar epithelial layer in bovine PCLS infected with different *Mmm* strains. Figure S7. Bronchiolar epithelial barrier integrity of bovine PCLS with and without infection. Figure S8. Determination of doubling times (Td) of *Mmc* GM12 and *Mmm* Afadé. Figure S9. Growth curves of *Mmc* GM12 and *Mmm* Afadé in cell culture (RPMI) medium. Supplementary video 1. A representative video showing full ciliary activity of PCLS.
