# Supplementary material for: Host-Pathogen Interactions of Mycoplasma mycoides in Caprine and Bovine Precision-Cut Lung Slices (PCLS) Models
Source: Pathogens. 2019 Jun 20;8(2):82. doi: 10.3390/pathogens8020082 (PMC6631151; doi:10.3390/pathogens8020082)
Supplement: Supplementary file 1 [file pathogens-08-00082-s001.zip › pathogens-526576-SI/Supplementary Materials/Supplementary materials.pdf]

Supplemental materials

Figure S1

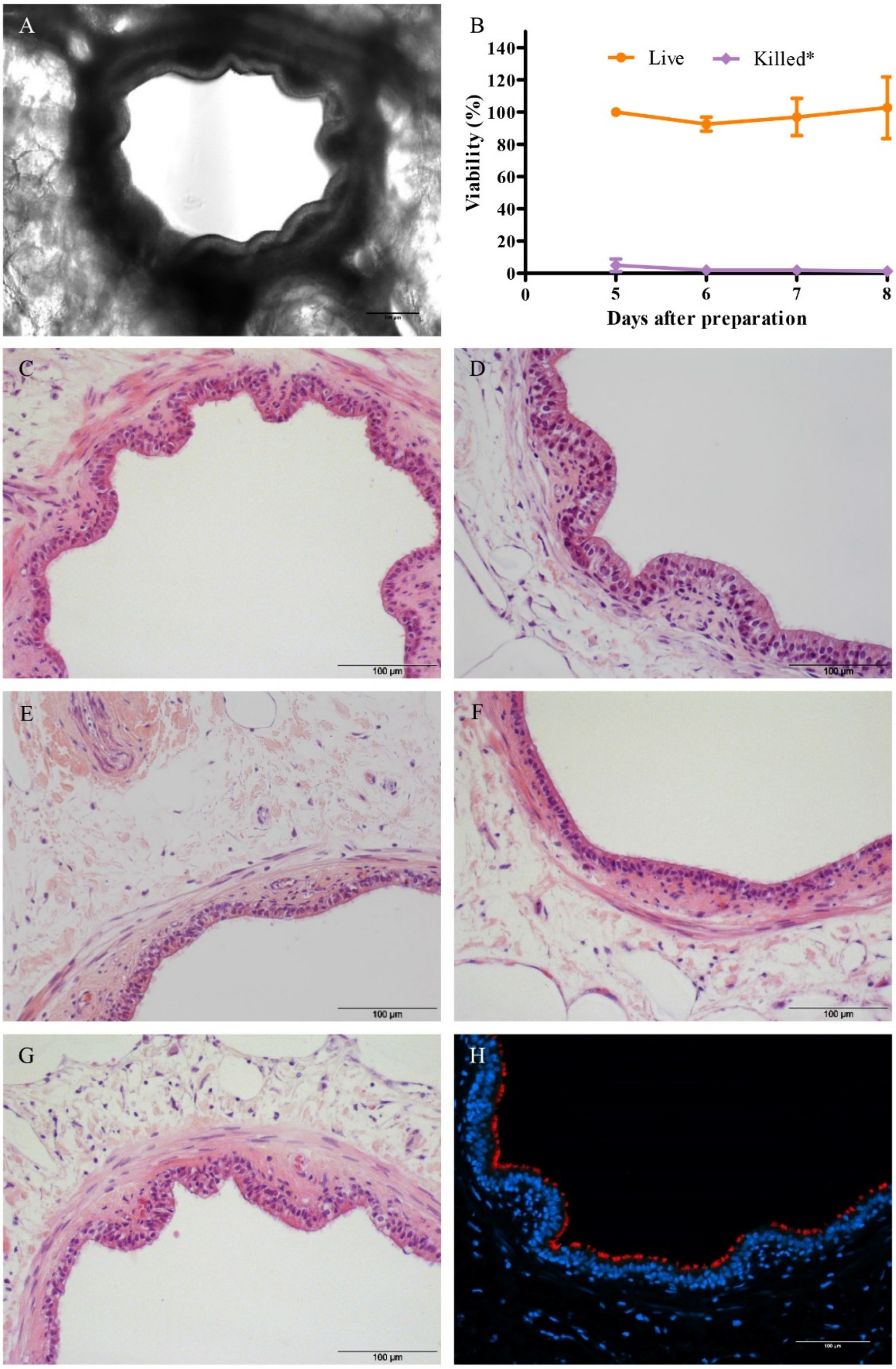

Figure S1. Quality control of PCLS.

Bronchiole under light microscope **(A)**; Percentage viability of PCLS using MTS Assay **(B)**; H&E staining at 4, 5, 6, 7, and 8 days after PCLS preparation (**C-G**, respectively); Immunofluorescence staining: red- β-tubulin of ciliated cells and blue- nuclei stained with DAPI **(H)**. During the entire duration of the experiment, the slices remained intact and viable. Red- β-tubulin of ciliated cells; Green- *Mmc*; Blue- Nuclei of caprine cells. Scale bars: 100 µm.

*PCLS samples were treated with 0.1 % Triton as a 100 % killed control in B.

Figure S2 A

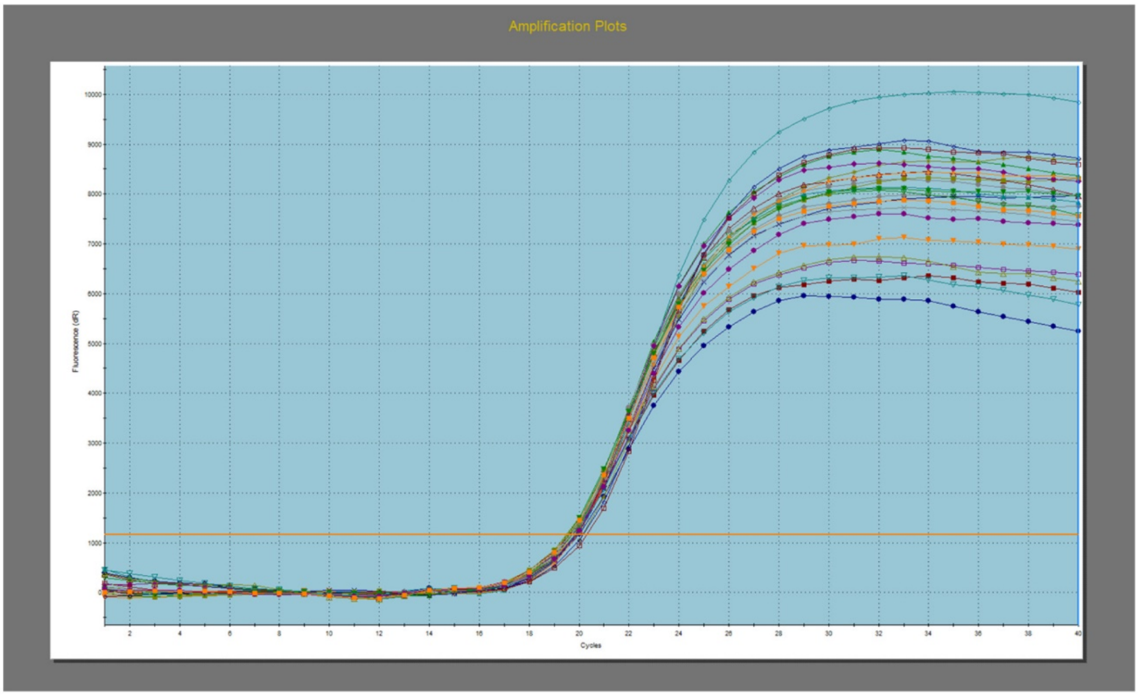

No.	Well Name	Dye	Assay	Ct (dR)
1	*Mmm* Afadé; 24h-1	SYBR	oCC18z	20.18
2	*Mmm* Afadé; 24h-2	SYBR	oCC18z	20.36
3	*Mmm* Afadé; 48h-1	SYBR	oCC18z	19.58
4	*Mmm* Afadé; 48h-2	SYBR	oCC18z	19.71
5	*Mmm* Afadé; 72h-1	SYBR	oCC18z	19.77
6	*Mmm* Afadé; 72h-2	SYBR	oCC18z	19.91
7	*Mmm* Afadé; 96h-1	SYBR	oCC18z	19.88
8	*Mmm* Afadé; 96h-2	SYBR	oCC18z	19.61
9	Uninfected control; 24h-1	SYBR	oCC18z	19.98
10	Uninfected control; 24h-2	SYBR	oCC18z	19.64
11	Uninfected control; 48h-1	SYBR	oCC18z	20.16
12	Uninfected control; 48h-2	SYBR	oCC18z	19.93
13	Uninfected control; 72h-1	SYBR	oCC18z	19.94
14	Uninfected control; 72h-2	SYBR	oCC18z	19.88
15	Uninfected control; 96h-1	SYBR	oCC18z	19.53
16	Uninfected control; 96h-2	SYBR	oCC18z	19.84
17	*Mmc* GM12; 24h-1	SYBR	oCC18z	19.65
18	*Mmc* GM12; 24h-2	SYBR	oCC18z	19.76
19	*Mmc* GM12; 48h-1	SYBR	oCC18z	19.78
20	*Mmc* GM12; 48h-2	SYBR	oCC18z	19.85
21	*Mmc* GM12; 72h-1	SYBR	oCC18z	19.77
22	*Mmc* GM12; 72h-2	SYBR	oCC18z	19.67
23	*Mmc* GM12; 96h-1	SYBR	oCC18z	19.91
24	*Mmc* GM12; 96h-2	SYBR	oCC18z	19.95

Figure S2 B

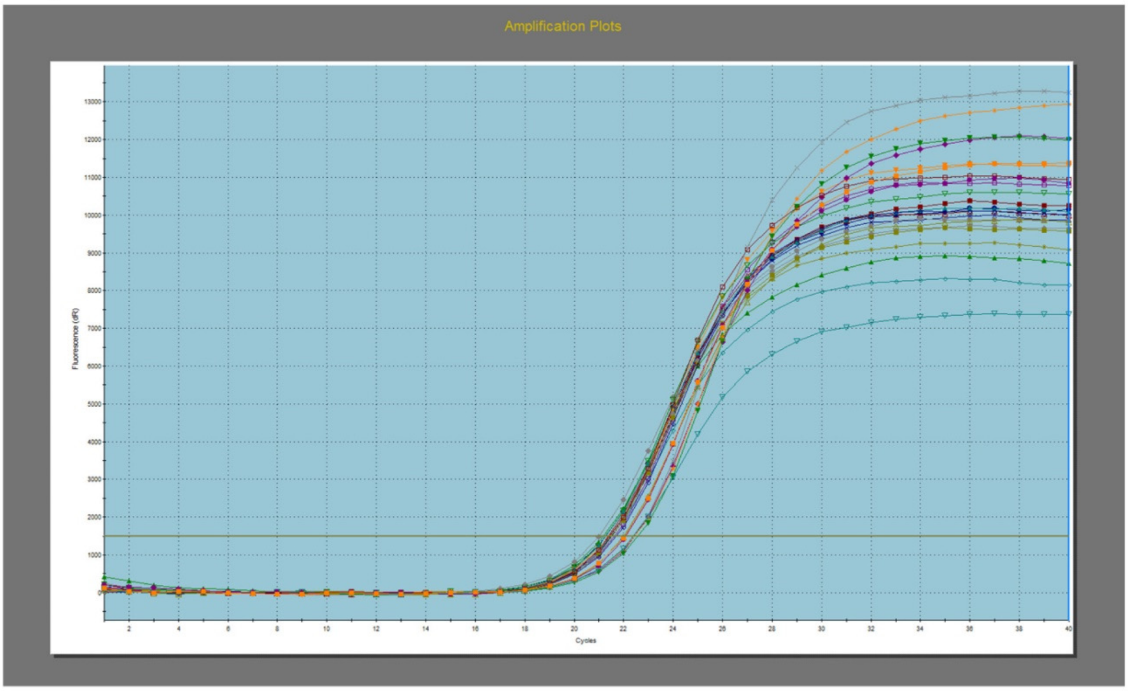

No.	Well Name	Dye	Assay	Ct (dR)
1	*Mmm* Afadé; 24h-1	SYBR	oCC18	21.58
2	*Mmm* Afadé; 24h-2	SYBR	oCC18	21.52
3	*Mmm* Afadé; 48h-1	SYBR	oCC18	22.01
4	*Mmm* Afadé; 48h-2	SYBR	oCC18	22.45
5	*Mmm* Afadé; 72h-1	SYBR	oCC18	21.32
6	*Mmm* Afadé; 72h-2	SYBR	oCC18	21.51
7	*Mmm* Afadé; 96h-1	SYBR	oCC18	22.11
8	*Mmm* Afadé; 96h-2	SYBR	oCC18	22.06
9	Uninfected control; 24h-1	SYBR	oCC18	21.32
10	Uninfected control; 24h-2	SYBR	oCC18	21.45
11	Uninfected control; 48h-1	SYBR	oCC18	21.6
12	Uninfected control; 48h-2	SYBR	oCC18	21.34
13	Uninfected control; 72h-1	SYBR	oCC18	21.23
14	Uninfected control; 72h-2	SYBR	oCC18	21.02
15	Uninfected control; 96h-1	SYBR	oCC18	22.51
16	Uninfected control; 96h-2	SYBR	oCC18	22.51
17	*Mmc* GM12; 24h-1	SYBR	oCC18	21.34
18	*Mmc* GM12; 24h-2	SYBR	oCC18	21.57
19	*Mmc* GM12; 48h-1	SYBR	oCC18	21.75
20	*Mmc* GM12; 48h-2	SYBR	oCC18	21.49
21	*Mmc* GM12; 72h-1	SYBR	oCC18	21.68
22	*Mmc* GM12; 72h-2	SYBR	oCC18	21.48
23	*Mmc* GM12; 96h-1	SYBR	oCC18	22.63
24	*Mmc* GM12; 96h-2	SYBR	oCC18	22.45

Figure S2. Comparison of cell numbers in caprine and bovine PCLS

Amplification plots from a qRT-PCR for caprine PCLS **(A)** and bovine PCLS **(B)** using caprine and bovine CEACAM18 genes as targets, respectively. Amplification plots include duplicates of uninfected control, *Mmm* Afadé and *Mmc* GM12 at 24, 48, 72, and 96 h p.i. Similar Ct values of all the tested samples indicate comparably similar number of caprine **(A)** and bovine **(B)** cells in each PCLS. The tables under each figure show the actual threshold cycle (Ct) values of the samples.

Figure S3

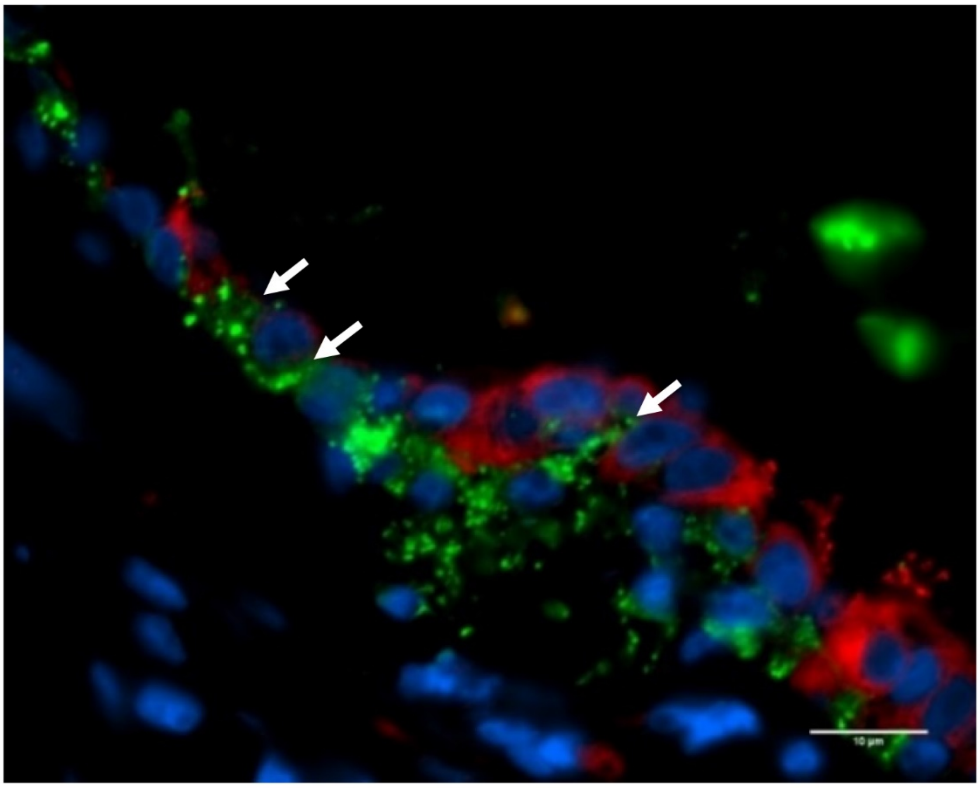

Figure S3. Paracellular localization of *Mmc* GM12 in caprine PCLS (96 h p.i.).
White arrows indicate *Mmc* GM12 "between" the bronchiolar epithelial cells. Red- β-tubulin of ciliated cells; Green- *Mmc*; Blue- Nuclei of caprine cells. Scale bar: 10 µm.

Figure S4

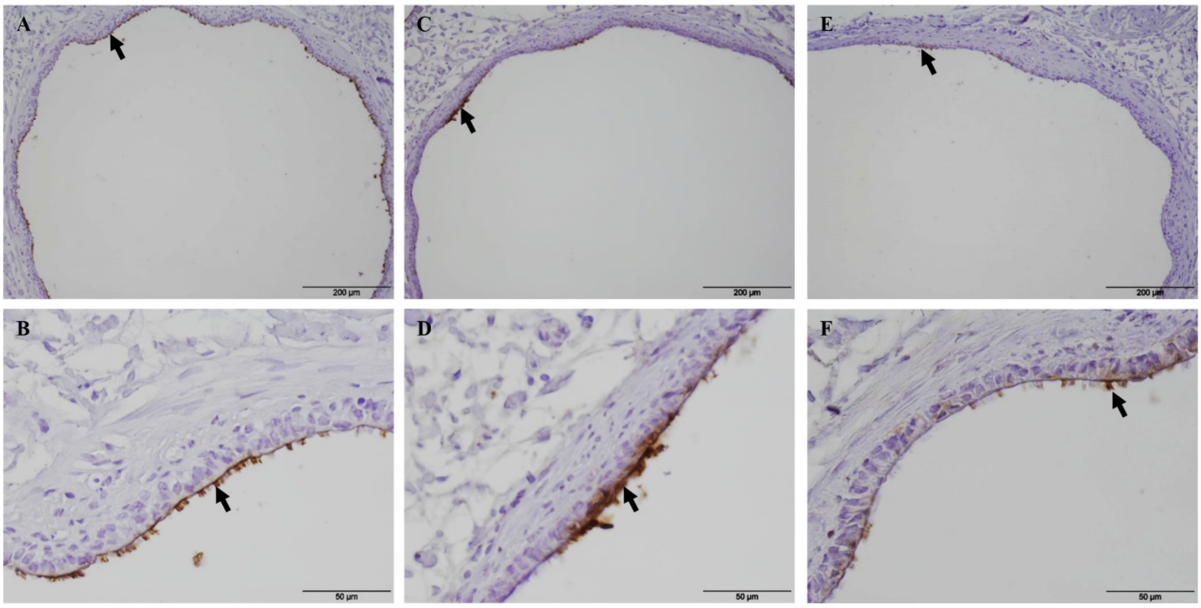

Figure S4. Adherence of *Mmm* Afadé to bovine ciliated epithelial cells (IHC).
Adherence of *Mmm* Afadé to the ciliated epithelial cells in bovine infected with the first experimental setup, where PCLS were infected for 4 hours and unbound bacteria were washed at 4 h p.i. Samples were further incubated with washing and medium change every 24 h p.i. IHC of bovine PCLS 48 (**A-B**, black arrows), 72 (**C-D**, black arrows) and 96 (**E-F**, black arrows) h p.i. Scale bars: A, C, E = 200 µm; B, D, F= 50 µm. Samples were stained with anti-*Mmm* PG1 primary antibody and DAKO Envision kit.

Figure S5

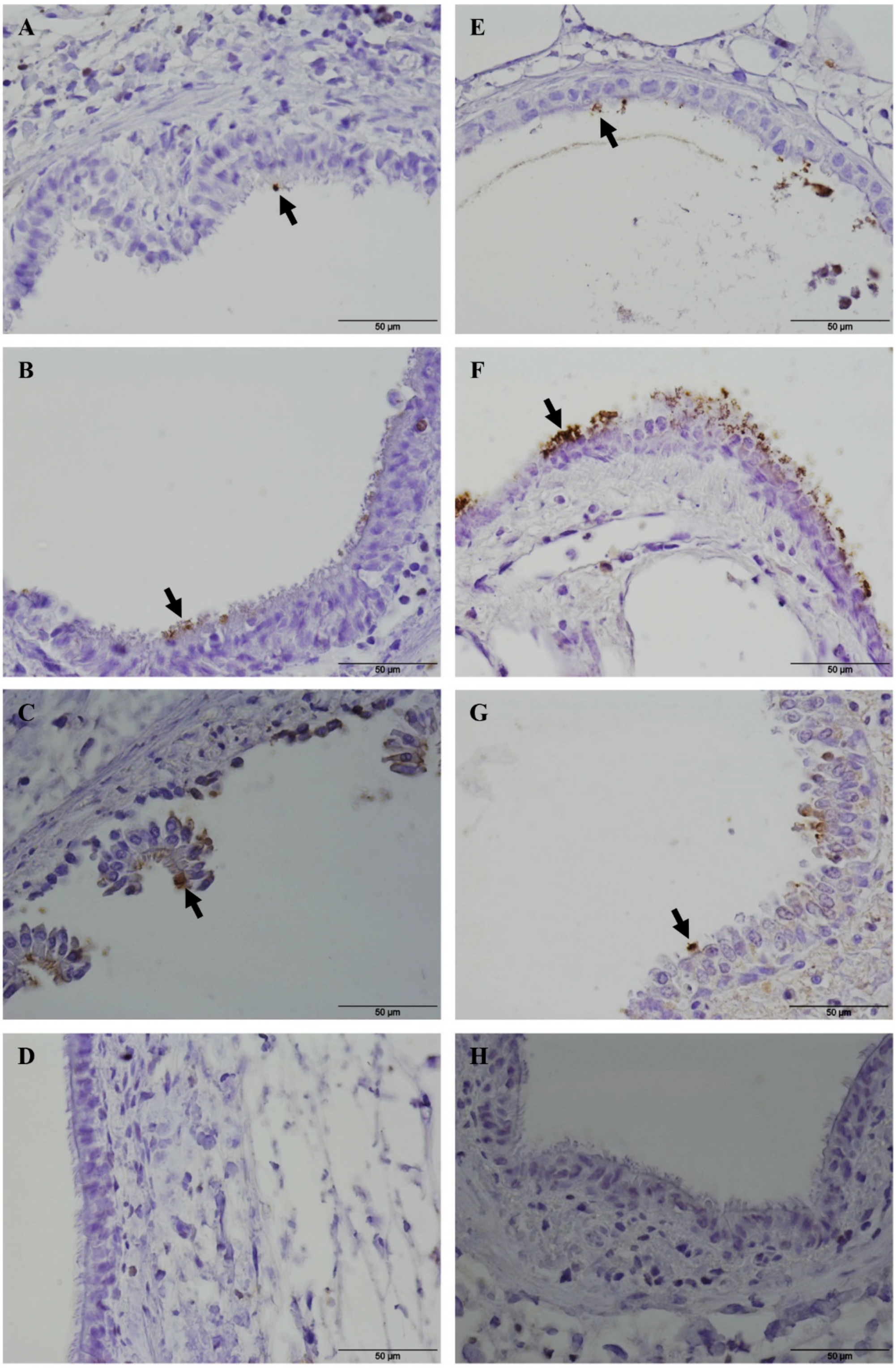

Figure S5. Stage of Tissue destruction in caprine and bovine PCLS (IHC).

Tissue destruction in caprine PCLS infected with *Mmc* GM12 **(A-D)** and bovine PCLS infected with *Mmm* Afadé **(E-H)** with the second experimental approach, where PCLS were continuously infected and samples evaluated after a continuous 4 **(A and E)**, 8 **(B and F)** and 24 **(C and G)** hours infection without washing in between. Uninfected controls at 24 h p.i in caprine **(D)** and bovine **(H)** PCLS are included for comparison. Bacterial adherence to the ciliated cells increased in a time dependent manner (black arrows) and finally resulted in the sloughing-off of the columnar epithelial layer from the remaining basal epithelium at 24 h p.i (C and G). All samples including uninfected controls were stained in the same way as figure S4.

Figure S6

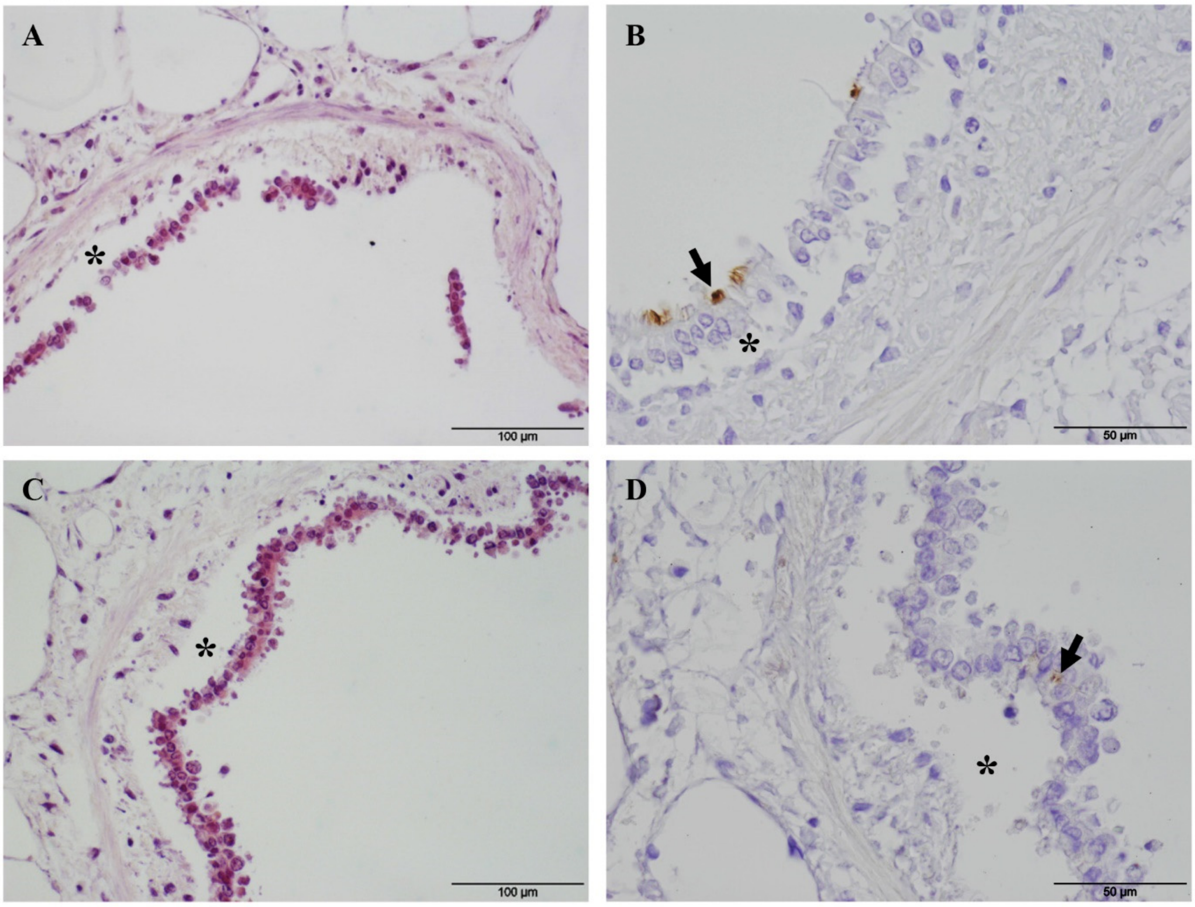

Figure S6. Destruction of the bronchiolar epithelial layer in bovine PCLS infected with different *Mmm* strains.

Tissue destruction following infection of bovine PCLS with *Mmm* B237 **(A and B)** and *Mmm* Gladysdale **(C and D)** for continuous 24 hours resulted in extensive detachment and destruction of the bronchiolar epithelial layer similar to the results observed in infection with *Mmm* Afadé and *Mmc* GM12. Bacteria were mainly adherent to the cilia (**B and D**, black arrows) and detachment of the upper part of the epithelium was observed in both strains 24 h p.i. (asterisk). A and C, H&E staining; B and D, IHC staining. Scale bars: A and C= 100 µm; B and D= 50 µm. IHC staining was conducted in the same way as figure S4.

Figure S7

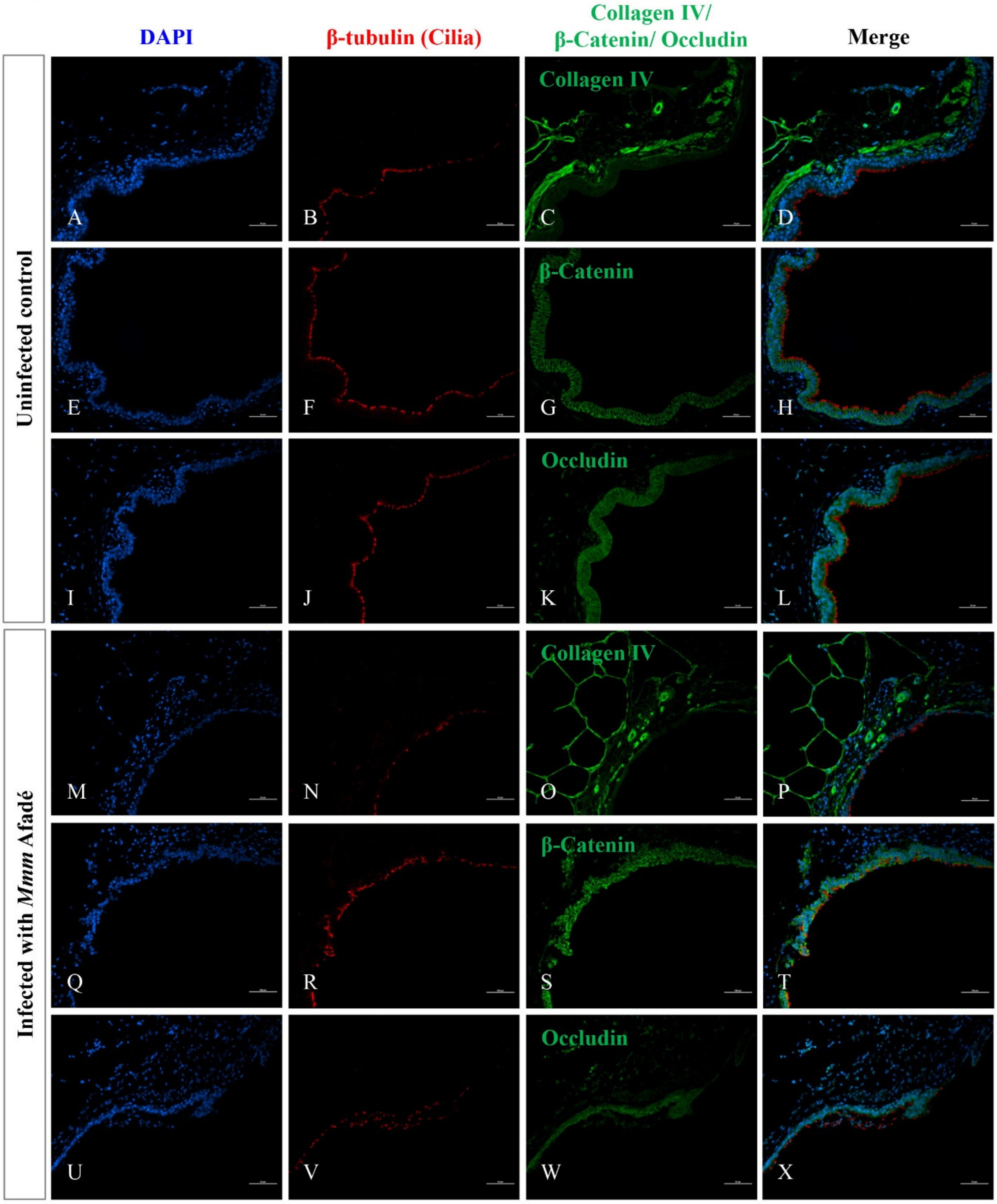

Figure S7. Bronchiolar epithelial barrier integrity of bovine PCLS with and without infection. Bronchiolar epithelial barrier integrity of uninfected bovine PCLS is demonstrated by immunofluorescence staining of PCLS sections **(A-L)**. After 24 h continuous infection with *Mmm* Afadé, loss of integrity is observed **(M-X)**. Nuclei of bovine cells were counterstained with DAPI (**A, E, I, M, Q, U,** blue) and cilia were labeled with a mouse monoclonal anti-β-tubulin–Cy3 antibody **(B, F, J, N, R, V,** red**)**. Staining of collagen IV **(C, O)**, β-catenin **(G, S)** and occludin **(K, W)**, as marker proteins for basement membrane, adherens and tight junctions, respectively. Primary antibodies against collagen IV, β-catenin, and occludin were stained using the corresponding Alexa Fluor 488 labeled secondary antibodies. Merged pictures illustrate the uninfected intact **(D, H, L)** and damaged bronchiolar epithelial cell layer following infection (P, T, X). Scale bars: 50 µm.

Figure S8

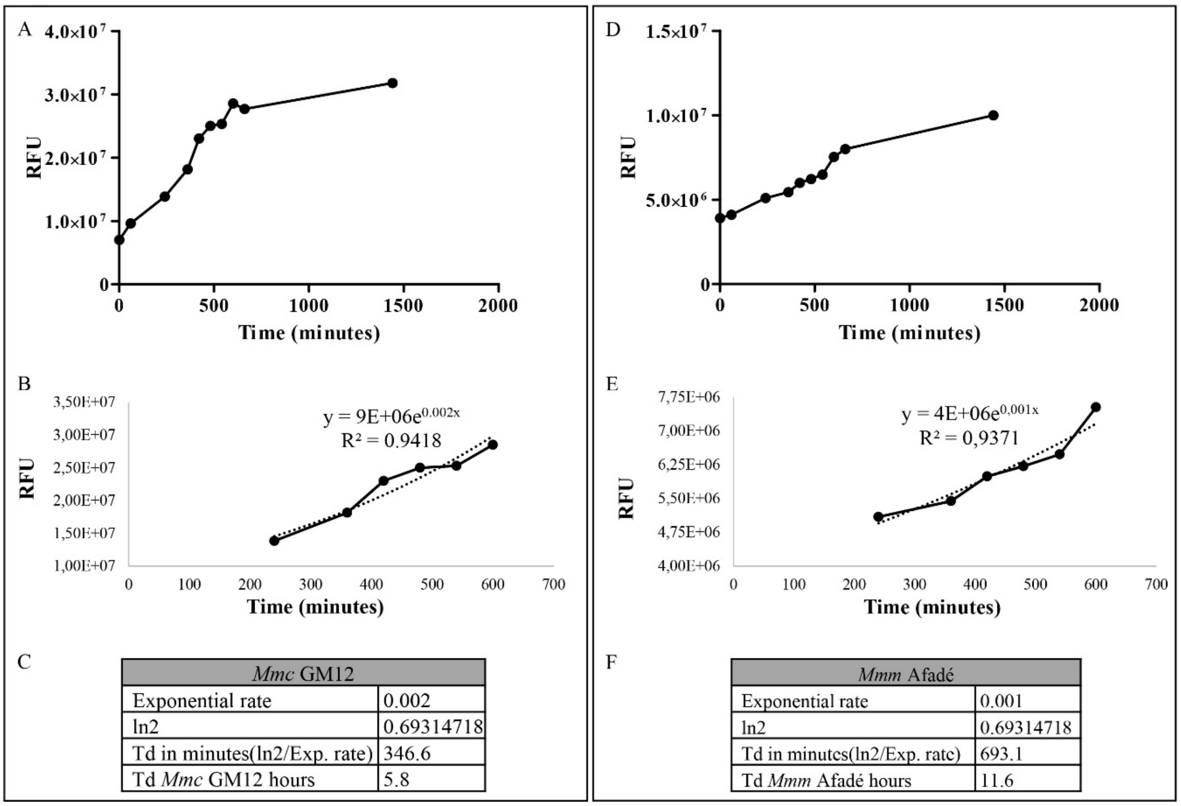

C

Mmc GM12	
Exponential rate	0.002
ln2	0.69314718
Td in minutes(ln2/Exp. rate)	346.6
Td *Mmc* GM12 hours	5.8

F

Mmm Afadé	
Exponential rate	0.001
ln2	0.69314718
Td in minutes(ln2/Exp. rate)	693.1
Td *Mmm* Afadé hours	11.6

Figure S8. Determination of doubling times (Td) of *Mmc* GM12 and *Mmm* Afadé.
Growth curves of *Mmc* GM12 **(A)** and *Mmm* Afadé **(D)** in RPMI-2 medium using Picogreen assay. Time points and equations used for the calculation of doubling times (Td) of *Mmc* GM12 **(B)** and *Mmm* Afadé **(E)**. Calculated Td of *Mmc* GM12 was 5.8 h **(C)**; and *Mmm* Afadé was 11.6 h **(F)** in RPMI-2 medium in the absence of PCLS; RFU-Relative fluorescence units.

Figure S9

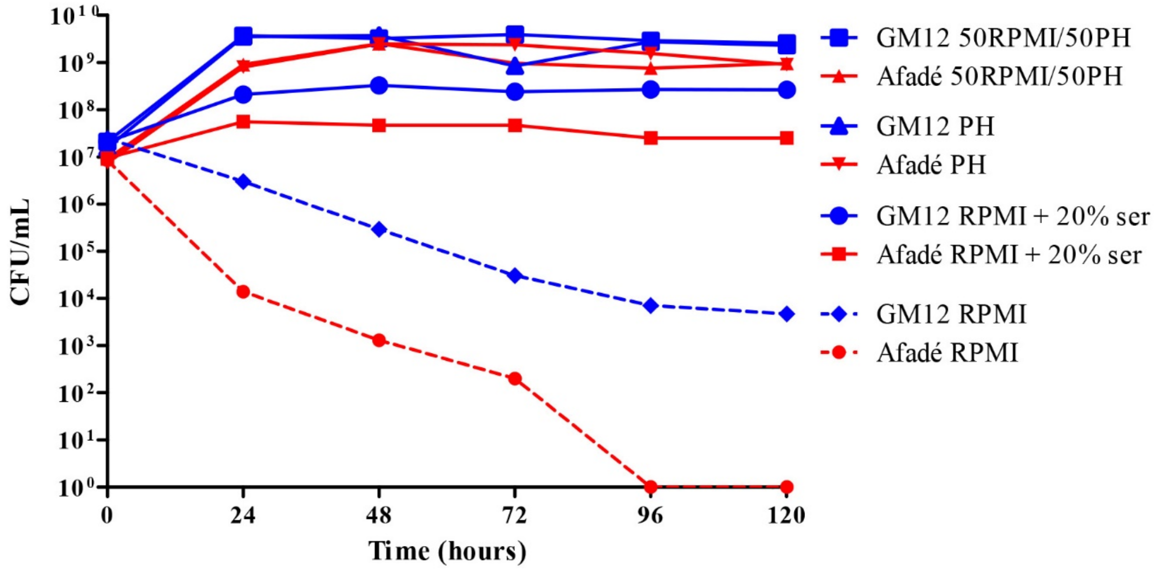

Figure S9. Growth curves of *Mmc* GM12 and *Mmm* Afadé in cell culture (RPMI) medium.
The ability of the cell culture medium to support the growth of *Mmc* GM12 and *Mmm* Afadé was tested. Different combinations including 20 % heat inactivated serum (RPMI-2) and standard PH medium were used. Both strains do not grow in the cell culture medium without the addition of serum. However, the addition of serum enhanced the growth of both strains. The mixture of RPMI and PH medium allowed comparable growth of the bacteria to the standard mycoplasma medium (PH medium). Accordingly the growths in 50RPMI/50PH = PH > RPMI + 20 % ser > RPMI medium. Note: 50RPMI/50PH- A medium composed of 50 % RPMI-2 medium and 50 % of PH medium containing 5 % heat inactivated bovine serum, which was used for infection of PCLS and maintaining uninfected controls.
Supplementary video 1. A representative video showing full ciliary activity of PCLS
