# Supplementary figures and images for: Host-Pathogen Interactions of Mycoplasma mycoides in Caprine and Bovine Precision-Cut Lung Slices (PCLS) Models
Source: Pathogens. 2019 Jun 20;8(2):82. doi: 10.3390/pathogens8020082 (PMC6631151; doi:10.3390/pathogens8020082)

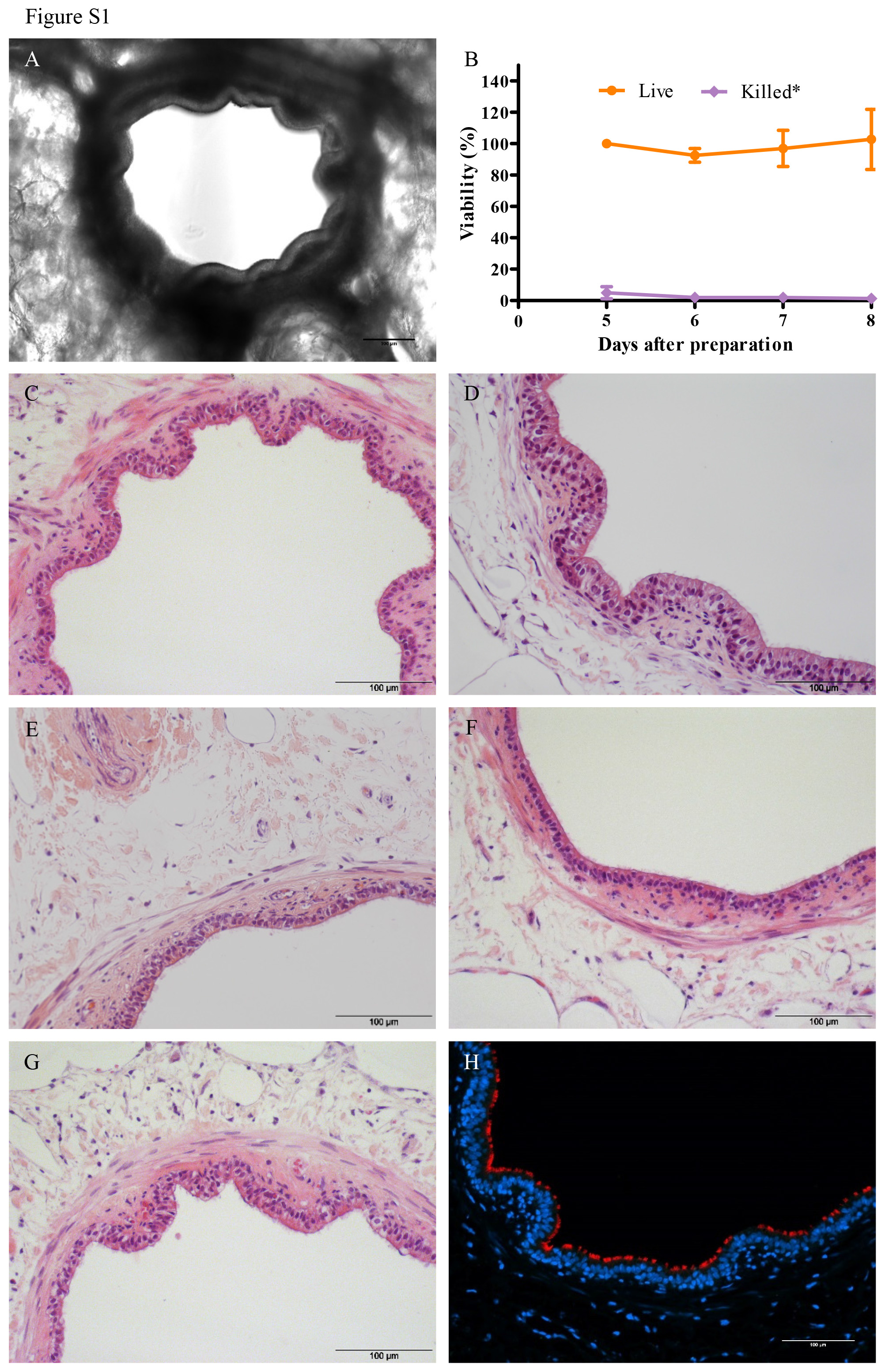

Supplement: Supplementary file 1 [file pathogens-08-00082-s001.zip › pathogens-526576-SI/Supplementary Materials/Figure S1.jpg]

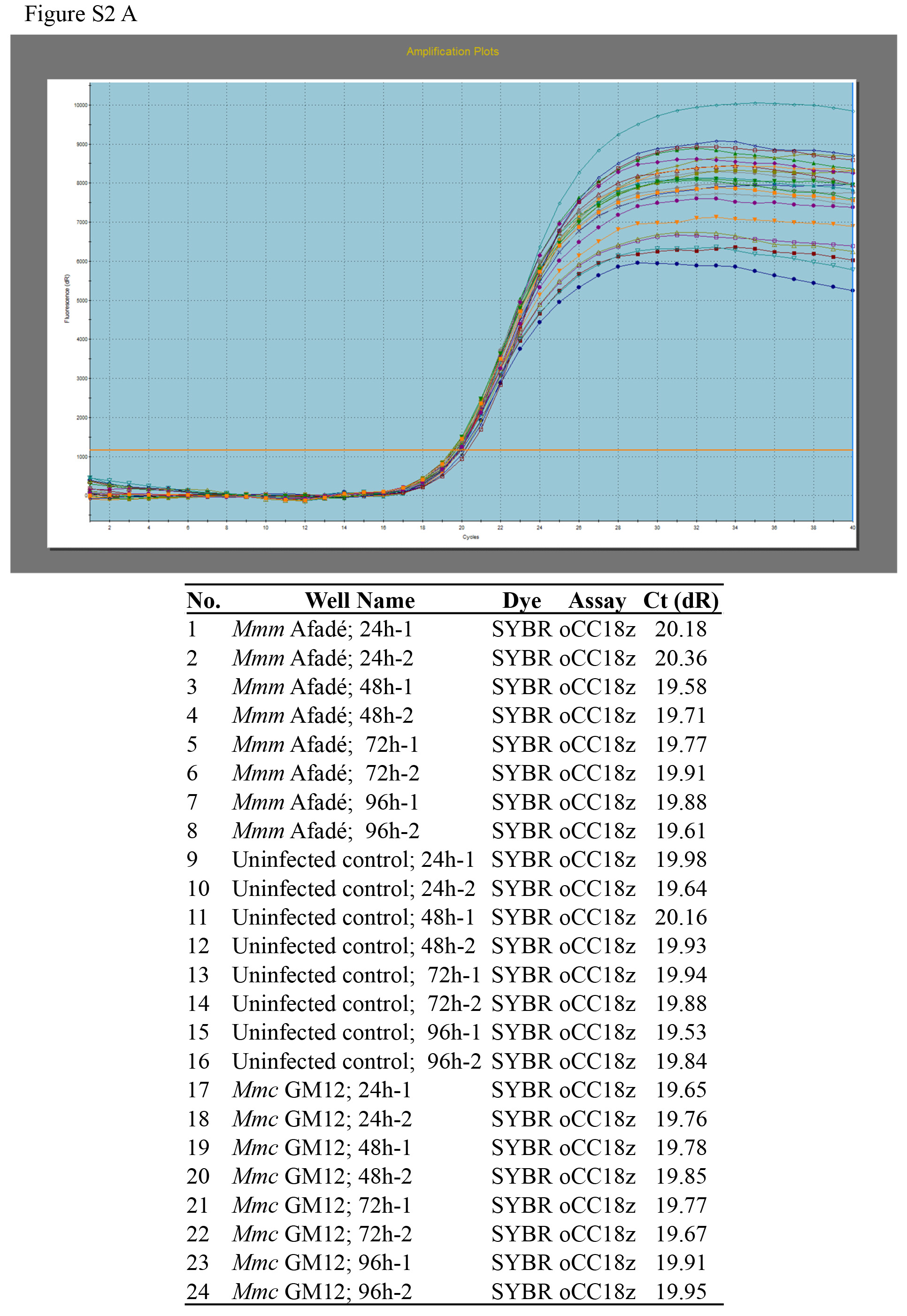

Supplement: Supplementary file 1 [file pathogens-08-00082-s001.zip › pathogens-526576-SI/Supplementary Materials/Figure S2A.jpg]

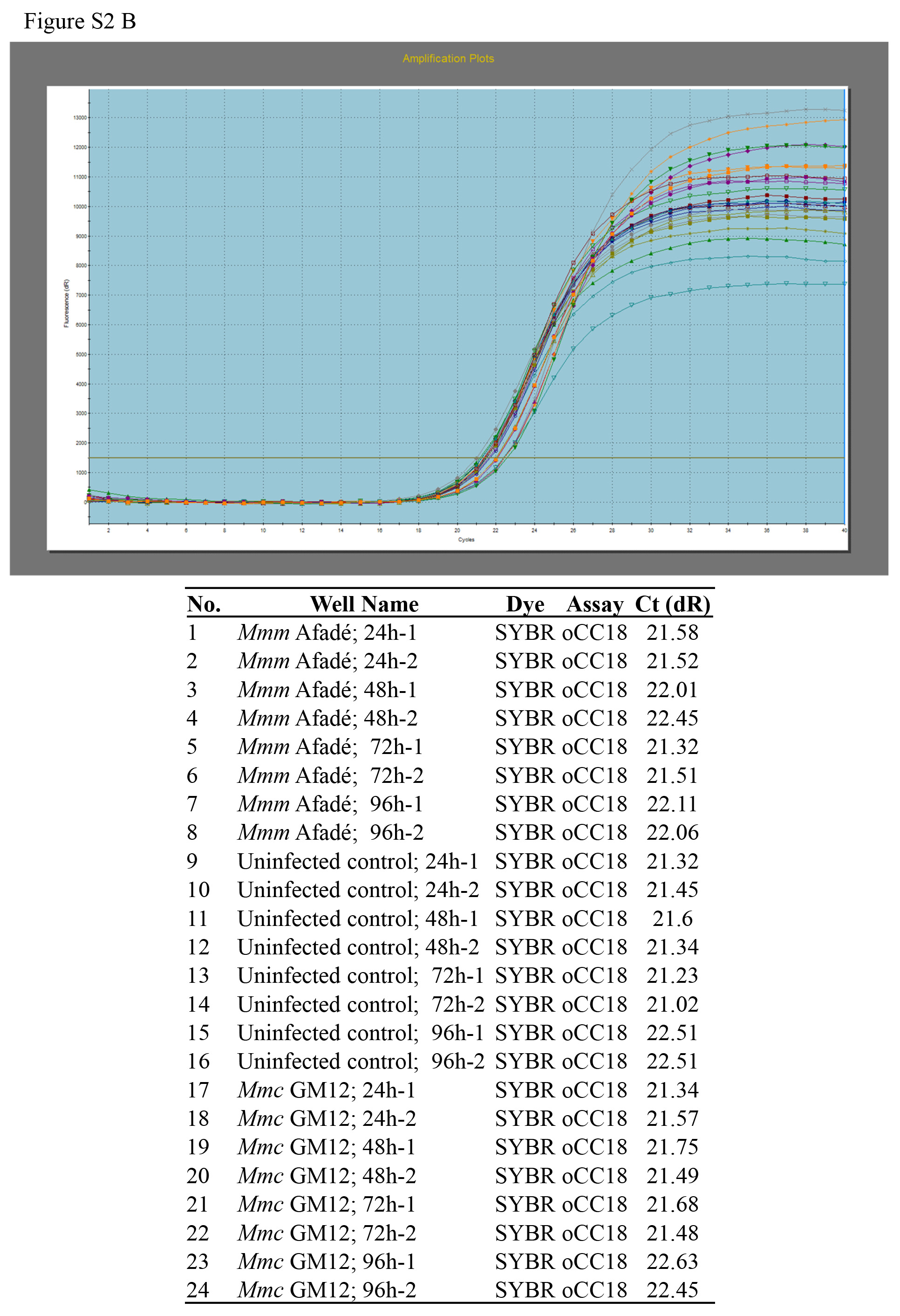

Supplement: Supplementary file 1 [file pathogens-08-00082-s001.zip › pathogens-526576-SI/Supplementary Materials/Figure S2B.jpg]

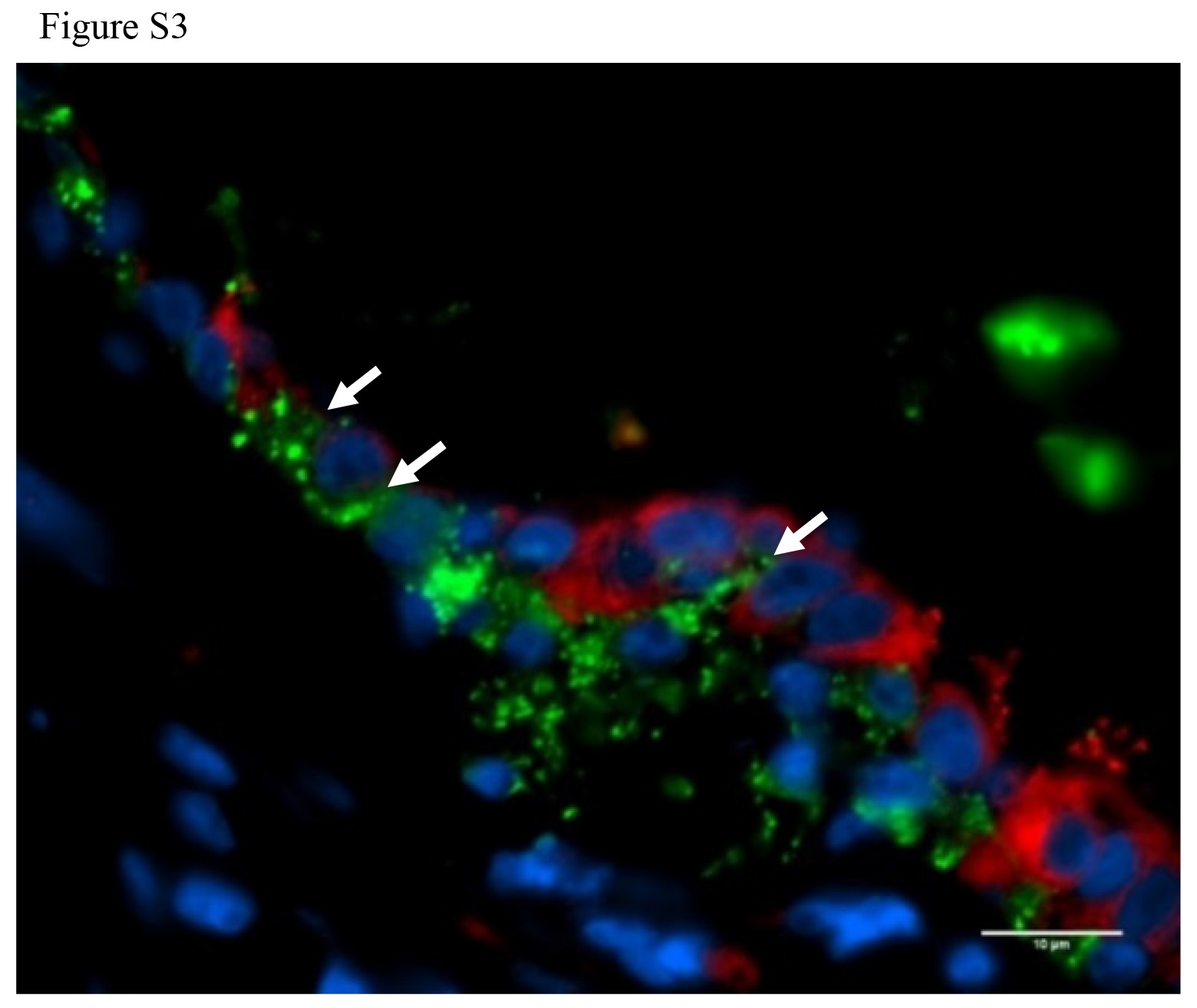

Supplement: Supplementary file 1 [file pathogens-08-00082-s001.zip › pathogens-526576-SI/Supplementary Materials/Figure S3.jpg]

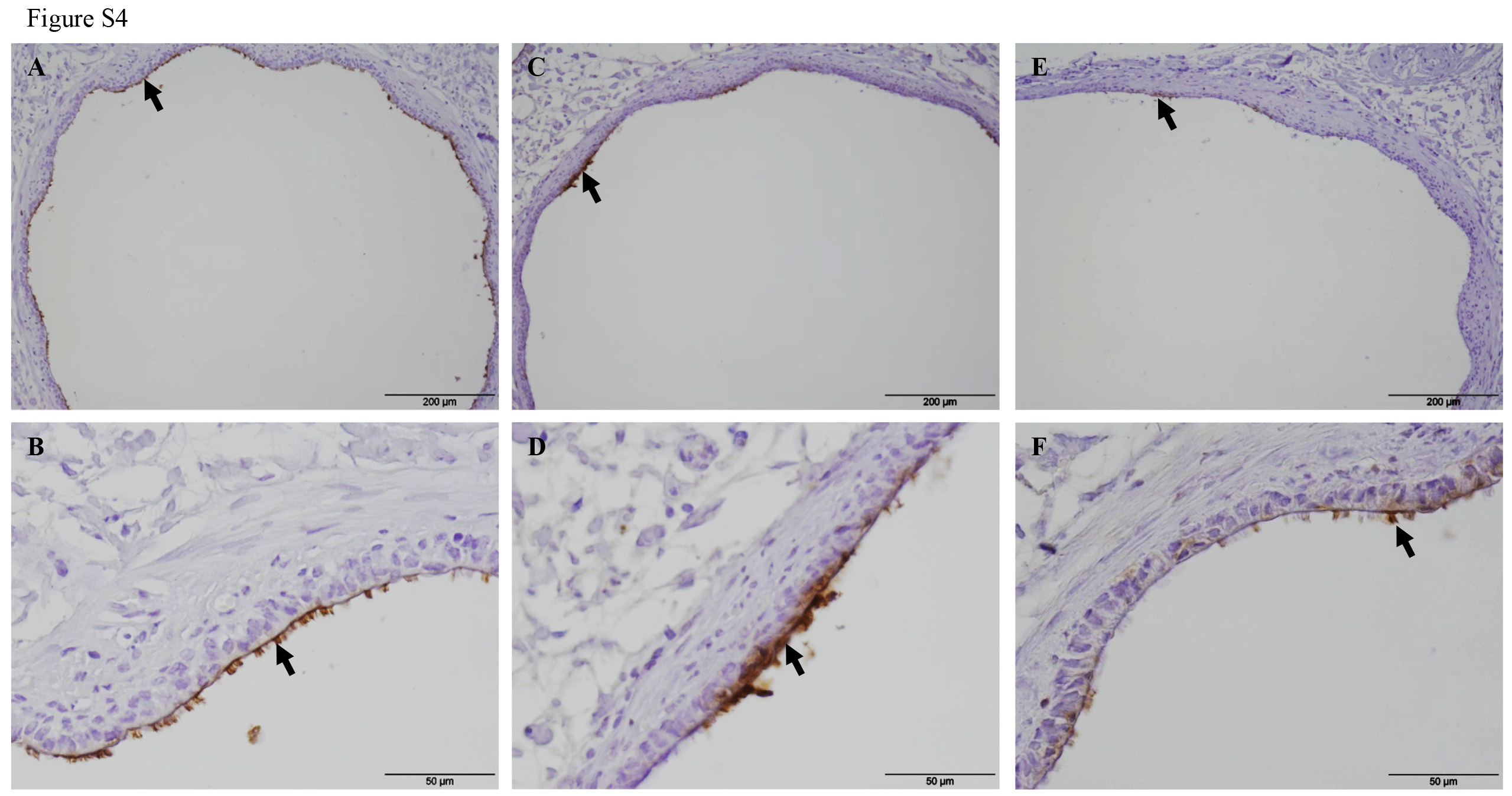

Supplement: Supplementary file 1 [file pathogens-08-00082-s001.zip › pathogens-526576-SI/Supplementary Materials/Figure S4.jpg]

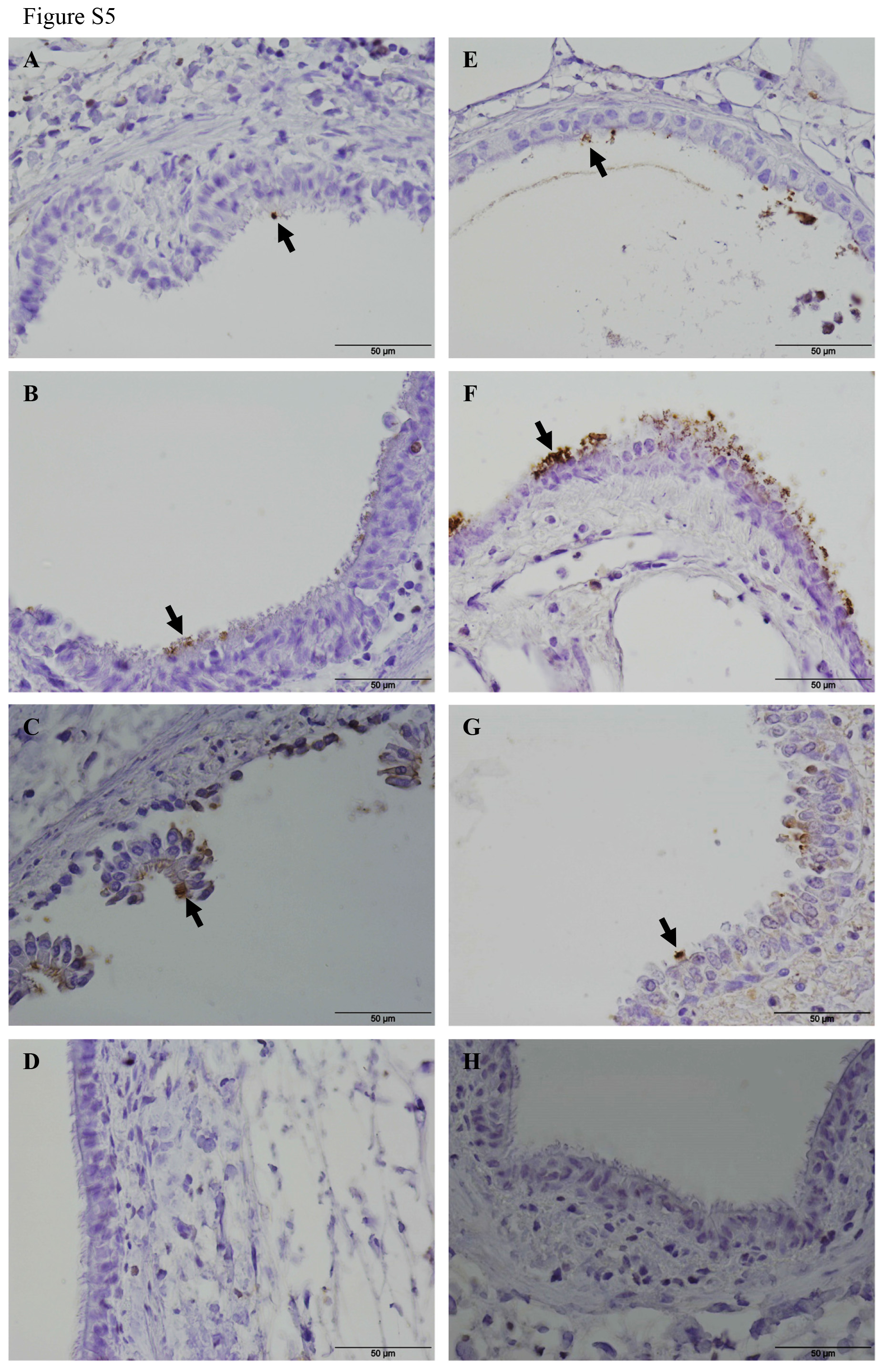

Supplement: Supplementary file 1 [file pathogens-08-00082-s001.zip › pathogens-526576-SI/Supplementary Materials/Figure S5.jpg]

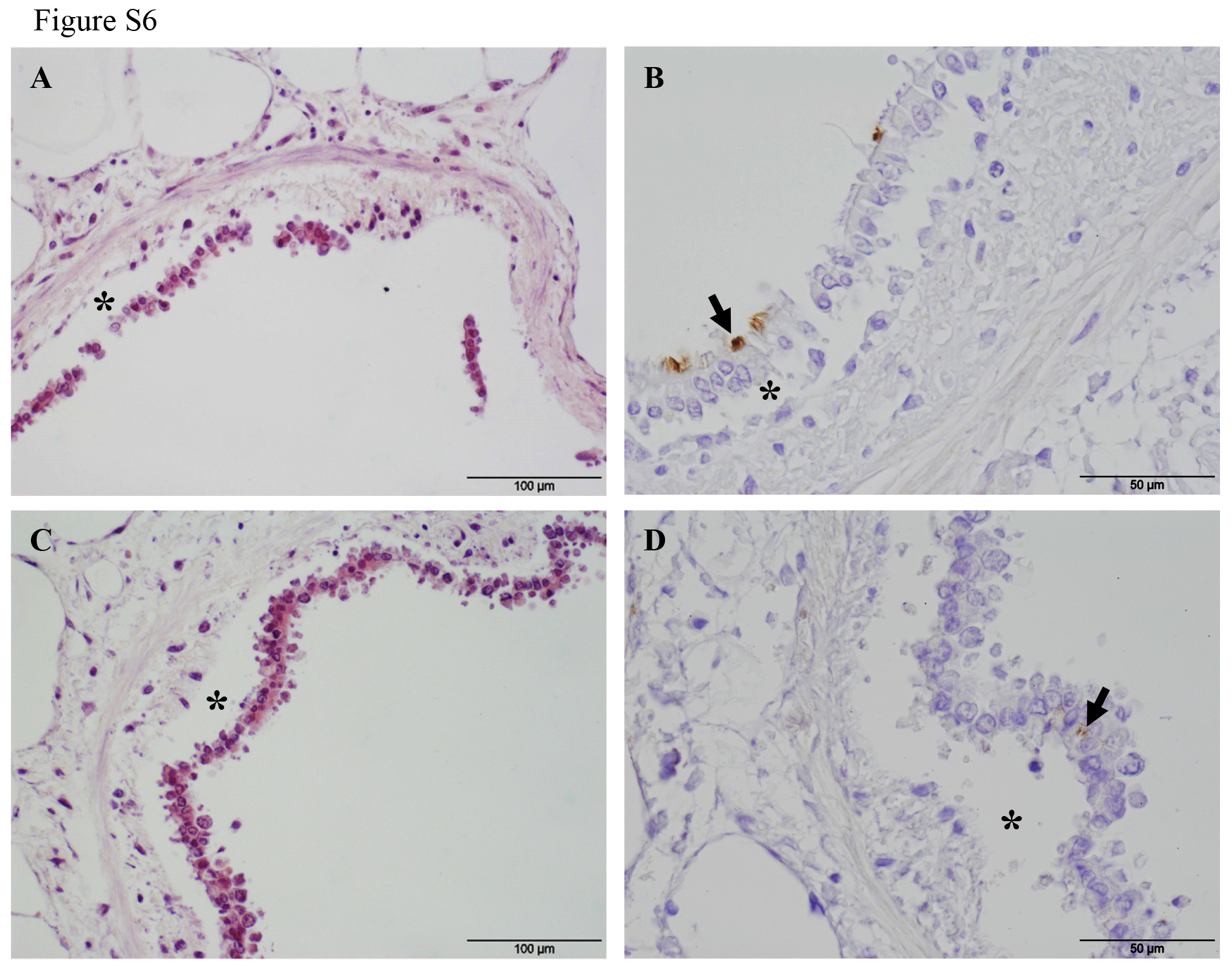

Supplement: Supplementary file 1 [file pathogens-08-00082-s001.zip › pathogens-526576-SI/Supplementary Materials/Figure S6.jpg]

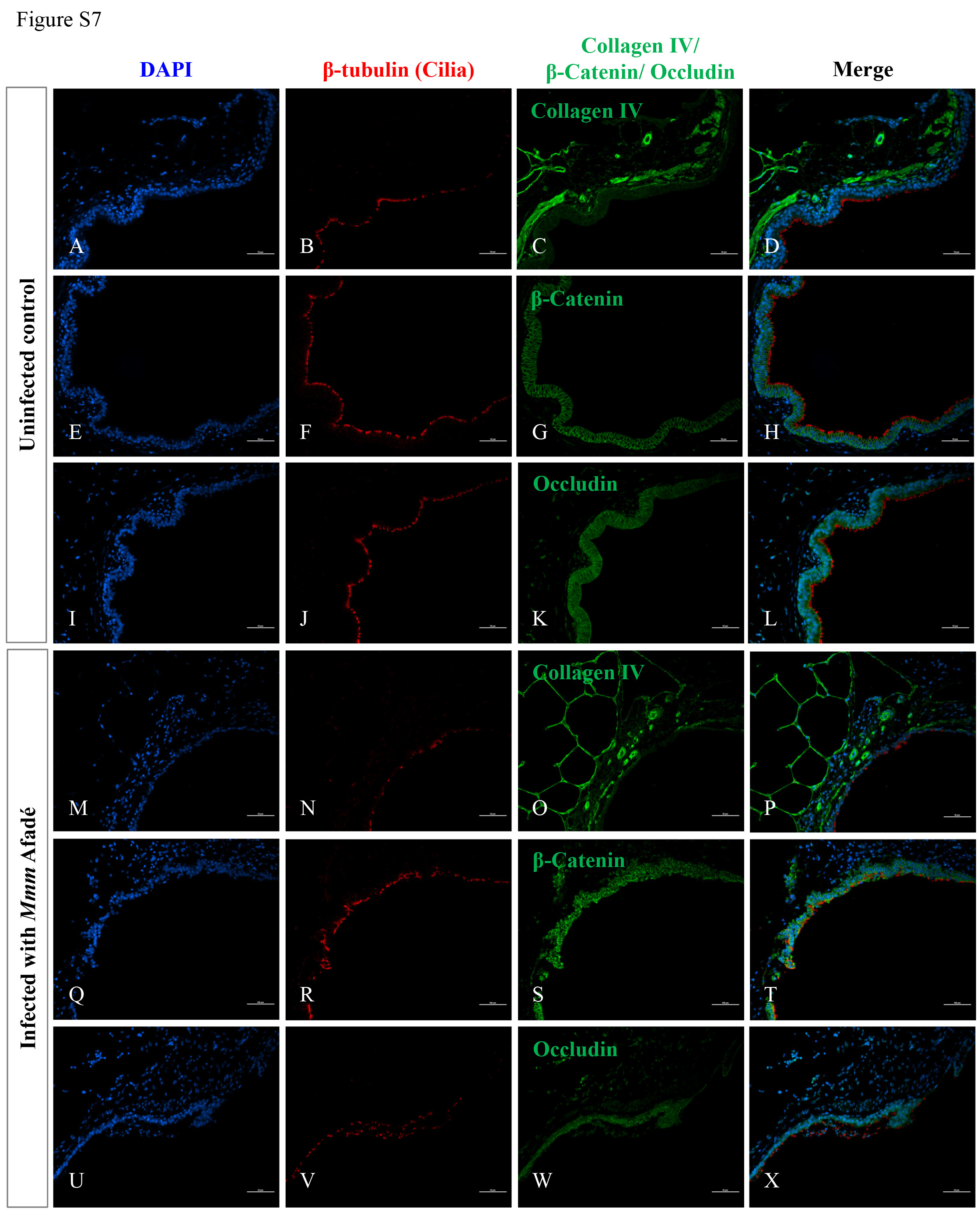

Supplement: Supplementary file 1 [file pathogens-08-00082-s001.zip › pathogens-526576-SI/Supplementary Materials/Figure S7.jpg]

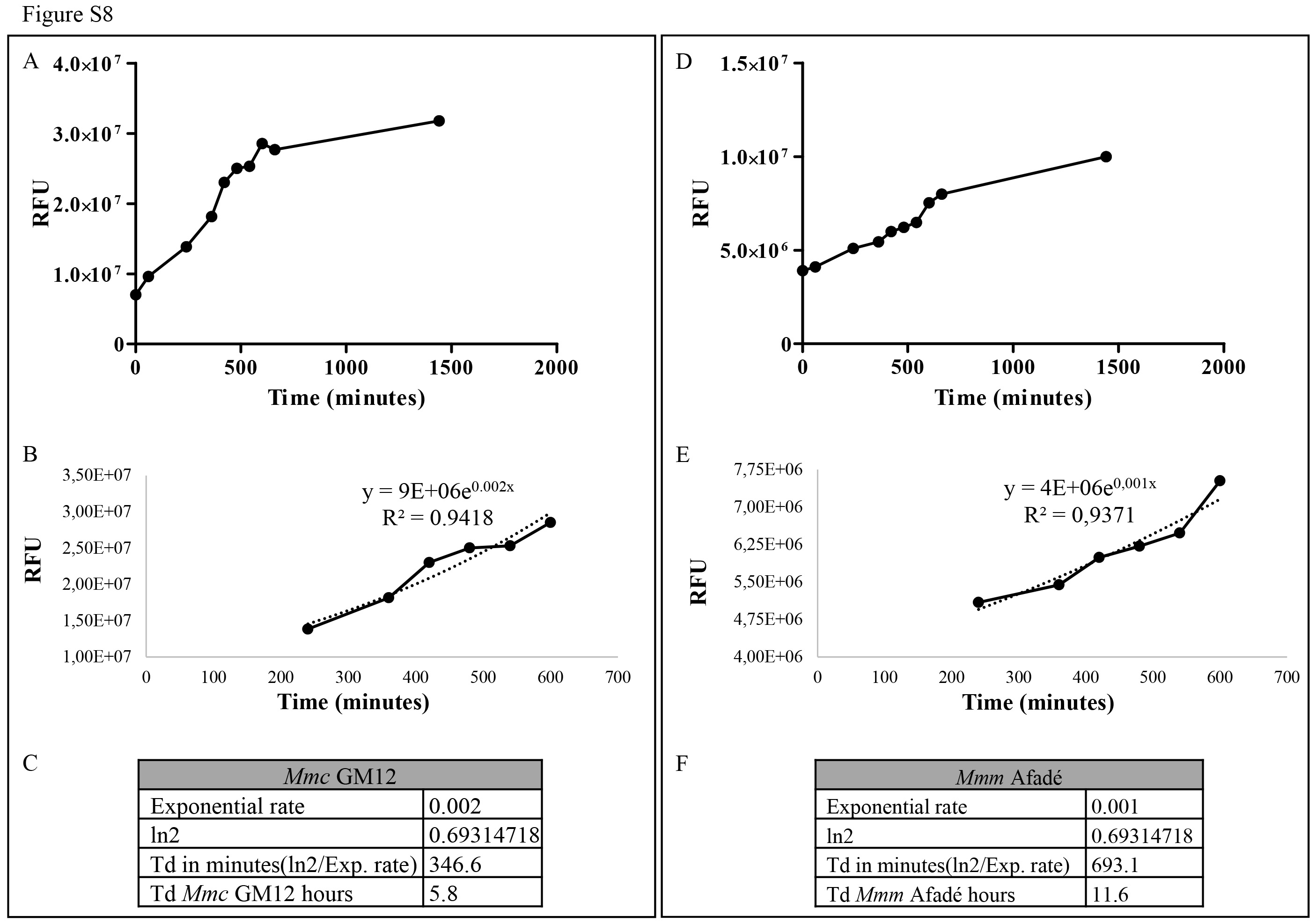

Supplement: Supplementary file 1 [file pathogens-08-00082-s001.zip › pathogens-526576-SI/Supplementary Materials/Figure S8.jpg]

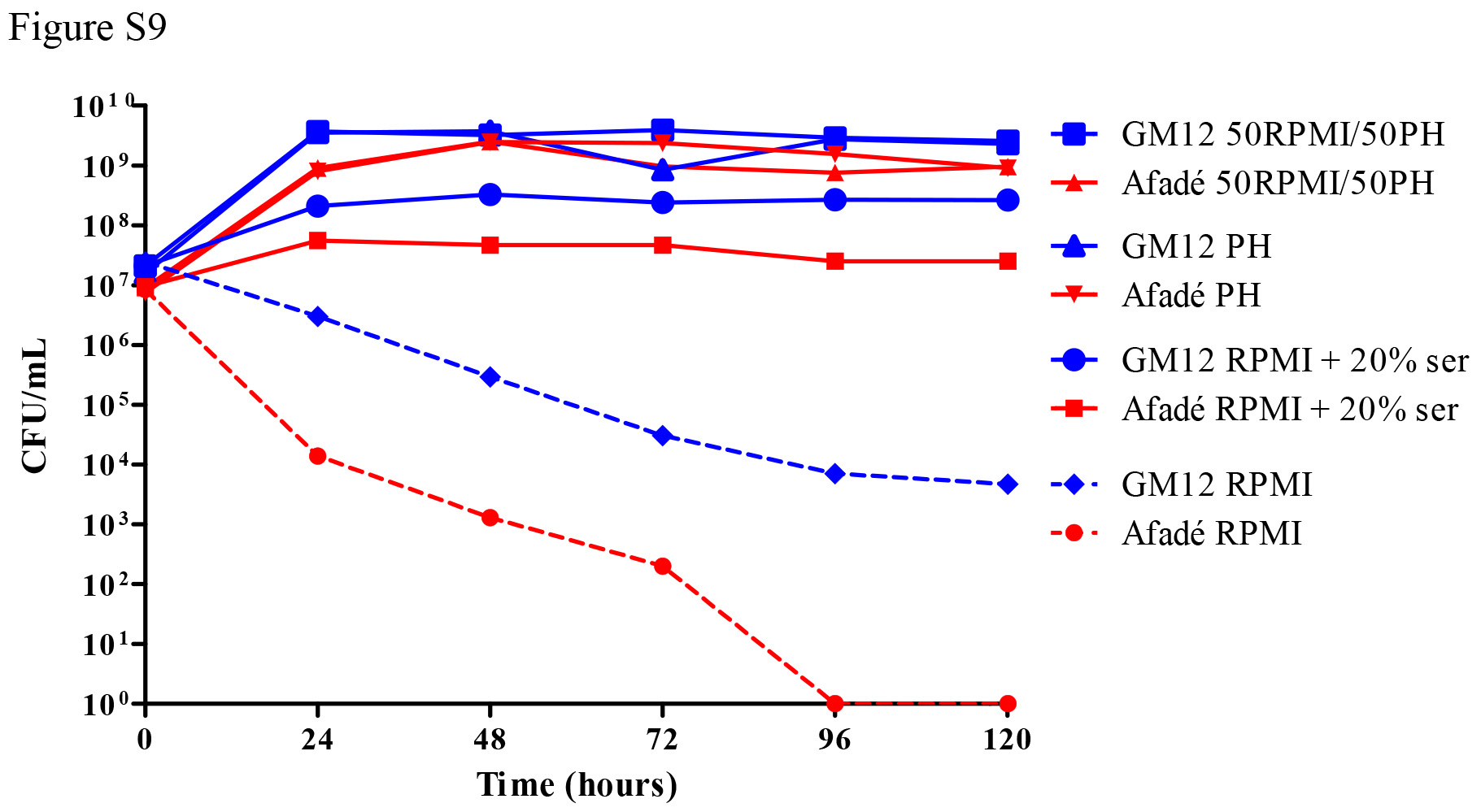

Supplement: Supplementary file 1 [file pathogens-08-00082-s001.zip › pathogens-526576-SI/Supplementary Materials/Figure S9.jpg]
